# An Updated Systematic Review and Meta-Analysis of Diagnostic Accuracy of Dynamic Contrast Enhancement and Diffusion-Weighted MRI in Differentiating Benign and Malignant Non-Mass Enhancement Lesions

**DOI:** 10.3390/jcm14134628

**Published:** 2025-06-30

**Authors:** Vera Nevyta Tarigan, Nungky Kusumaningtyas, Nina I. S. H. Supit, Edwin Sanjaya, Malvin Chandra, Callistus Bruce Henfry Sulay, Gilbert Sterling Octavius

**Affiliations:** 1 Breast and Female Reproductive Radiology, Department of Radiology, Faculty of Universitas Pelita Harapan, Tangerang 15811, Indonesia; 2 Department of Radiology, Siloam Hospital Kebon Jeruk, Jakarta 11530, Indonesia; 3 Department of Radiology, Siloam Hospital MRCCC, Jakarta 12930, Indonesia; 4 Radiology Resident, Faculty of Universitas Pelita Harapan, Tangerang 15811, Indonesia; 5 Department of Radiology, Siloam Hospital Lippo Village, Tangerang 15811, Indonesia

**Keywords:** magnetic resonance imaging, non-mass enhancement, diffusion-weighted imaging, dynamic contrast enhancement, apparent diffusion coefficient

## Abstract

**Objectives**: This study systematically evaluates the diagnostic accuracy of dynamic contrast-enhanced MRI (DCE-MRI), diffusion-weighted imaging (DWI), and apparent diffusion coefficient (ADC) values. **Methods**: The literature search started and ended on 10 June 2024. We searched MEDLINE, Cochrane Library, Pubmed, Science Direct, and Google Scholar. Our research question could be formulated as “In women with NME detected by MRI, how accurate are DCE and DWI in ruling in and ruling out malignancy when the diagnosis is compared to histopathology analysis with or without a clinical follow-up?”. The meta-analysis was conducted using the STATA 17 software with the “midas” commands. The study protocol has been registered in the International Prospective Register of Systematic Reviews (PROSPERO) database. **Results**: Fifty-four studies involving 6121 NME lesions were analyzed. The combined use of DCE-MRI and DWI demonstrated the highest diagnostic accuracy (AUC: 0.91; 95% CI: 0.88–0.93), followed by DWI alone (AUC: 0.85; 95% CI: 0.81–0.87) and ADC (AUC: 0.77; 95% CI: 0.74–0.81). DCE-MRI alone showed the lowest performance (AUC: 0.68; 95% CI: 0.64–0.72). Significant heterogeneity was observed across all modalities, with I^2^ values exceeding 95% in several analyses. The likelihood ratio scattergram indicated that no modality reliably confirmed or excluded malignancy. **Conclusions**: While the combination of DCE-MRI and DWI achieves the highest diagnostic accuracy, no modality can reliably differentiate benign from malignant NME lesions. Standardized imaging protocols and refined diagnostic descriptors are needed for clinical improvement.

## 1. Introduction

The use of breast magnetic resonance imaging (MRI) has been steadily increasing for diagnostic and screening purposes in clinical practice. While MRI is highly sensitive in detecting lesions, its specificity remains relatively low [1]. MRI combines high-resolution sequences to assess morphology with dynamic sequences to evaluate functionality. However, certain benign and malignant lesions can exhibit similar imaging characteristics. Given the higher prevalence of benign lesions compared with malignant ones, this overlap often results in more false-positive findings than true-positive results [2].

Non-mass enhancement (NME) refers to a region of enhancement observed on MRI that is not associated with a three-dimensional mass and lacks the defined characteristics of a mass [3]. NMEs can represent either benign or malignant lesions, with studies reporting that NMEs containing invasive components constitute approximately 10–42% of all malignant NMEs. Additionally, ductal carcinoma in situ (DCIS) has been shown to frequently present as NME, with reported rates ranging from 69% to 90% [4]. Hence, the differentiation between benign and malignant NMEs is crucial as it will affect clinical management.

No single imaging modality has been established as reliably effective for this purpose. Techniques such as magnetic resonance spectroscopy, proton magnetic resonance spectroscopy, intravoxel incoherent motion, non-Gaussian diffusion, positron emission tomography, and photoacoustic imaging have been proposed to aid differentiation; however, robust evidence remains lacking [5,6,7]. Consequently, dynamic contrast-enhanced (DCE)-MRI, diffusion-weighted imaging (DWI), and its quantitative parameter, the apparent diffusion coefficient (ADC), which are more readily available in clinical settings, are frequently utilized for evaluating NME lesions [8,9,10].

Two previous meta-analyses have addressed this topic. The first, published in 2013, utilized the 2003 version of the Breast Imaging Reporting and Data System (BI-RADS) [11]. Therefore, their findings may not reflect the current situation, especially with the advancement of MRI machines and protocols. The second, published in 2024 [12], included only thirteen studies on DCE-MRI and five on DWI, whereas the 2013 meta-analysis covered fifteen studies on DCE. From our perspective, significantly more studies have reported the diagnostic accuracy of DCE, DWI, and ADC, individually or in combination, than those included in these reviews, compromising their comprehensiveness. This systematic review and meta-analysis aims to evaluate the diagnostic accuracy of DCE-MRI, DWI, and ADC, individually and in combination, in distinguishing benign from malignant non-mass enhancement (NME). A secondary objective is to identify the DCE-MRI descriptors that most effectively differentiate between benign and malignant NME. This meta-analysis is also timely as the sixth BI-RADS edition will soon be updated.

## 2. Materials and Methods

### 2.1. Eligibility Criteria

The authors followed the Preferred Reporting Items for Systematic Review and Meta-Analysis of Diagnostic Test Accuracy (PRISMA-DTA) guidelines [13]. The pro-tocol for this study is available in the International Prospective Register of Systematic Reviews (PROSPERO) database (The PROSPERO code is CRD 42024554683, and the registration date is 4 June 2024).

The studied population was women with NME detected by MRI, especially in the enhanced sequences, which is defined as an area of enhancement containing non-enhancing fatty or glandular tissue distinct from a focus (a small punctate enhancement less than 5 mm) or a mass (an enhancement lesion larger than 5 mm in three dimensions that occupies space) [14]. The primary outcome of this study was to examine the sensitivity, specificity, positive likelihood ratio (LR+), negative likelihood ratio (LR−), positive predictive value (PPV), negative predictive value (NPV), and post-test probability of DCE and DWI in differentiating benign and malignant NME. The gold standard was histopathology analysis, with or without clinical follow-up. Hence, our research question could be formulated as “In women with NME detected by MRI, how accurate are DCE and DWI in ruling in and ruling out malignancy when the diagnosis is compared to histopathology analysis with or without a clinical follow-up?”

The inclusion criteria were articles of any cross-sectional, case–control cohort or randomized controlled trial published in any language. The search was also conducted for gray literature, including theses, dissertations, and conference abstracts. The exclusion criteria included reviews, case series, reports, and animal research. We excluded studies that only mentioned non-palpable breast masses without specifying the status of NME. Studies that did not provide enough data to calculate true positives (TP), false positives (FP), true negatives (TN), and false negatives (FN), even after contacting the authors for additional information, were excluded. Studies that utilized artificial intelligence without radiologists’ involvement would also be excluded. Finally, studies with fewer than 40 lesions were excluded because they could lead to unreliable results [15]. Review study citations were looked up to ensure that the literature was saturated. We manually searched and cited the literature to ensure all pertinent studies were covered.

### 2.2. Search Strategy and Study Selection

The literature search started and ended on 10 June 2024. We searched five academic databases: MEDLINE, Cochrane Library, Pubmed, Science Direct, and Google Scholar. The keywords used were related to the diagnostic tool (“magnetic resonance imaging”), anatomical site (“breast”), and the conditions under study (“non-mass,” “non-mass like,” “non-mass enhancement,” and “non-mass lesion”). Supplementary Table S1 lists each database’s Medical Subject Heading (MeSH) terms. We purposely avoided using any keywords that mentioned “sensitivity,” “specificity,” or any other terms connected to diagnosis because doing so could cause relevant research to be overlooked [16]. We also included the terms “diffusion-weighted imaging” and “dynamic contrast enhancement” to avoid missing any studies. All records were entered into the Rayyan program, which manually screened them and automatically identified duplicates [17]. Two authors (GSO and CBHS) conducted the first search and imported all the information into Rayyan software. Two different authors (MC and ES) cross-checked the initial searches. The four authors independently evaluated every paper. VNT, NS, and NK used group discussion and professional judgment to resolve conflicts. We chose the data that provided us with the greatest information when research from the same dataset had overlapping time points.

### 2.3. Data Extraction and Quality Assessment

Two authors (GSO and ES) independently extracted the data, while three (MC, CBHS, and VNT) verified its accuracy. We collected relevant data encompassing study identifiers (author and publication year), study attributes (such as location, design, participant age, use of blinded interpretation, involvement of breast radiologists, and study duration), MRI-related details (including magnet strength, manufacturer, and coil type), dynamic contrast enhancement parameters (scan sequences and orientation, number of dynamic phases, patient positioning, and slice thickness), diffusion metrics (apparent diffusion coefficient [ADC] values), and tumor characteristics (histopathological subtypes, counts of benign and malignant lesions, and lesion size and diameter).

The distribution patterns assessed included segmental, regional, linear focal, diffuse, and multifocal types. Four internal enhancement patterns were evaluated: homogeneous, heterogeneous, clustered ring, and clumped. Additionally, three kinetic enhancement curves were analyzed: plateau, persistent, and washout. Each of the definitions followed the BI-RADS nomenclature [18].

When articles included a category of “indeterminate” or “inconclusive” results, we would not extract the data on that category and focused only on the positive and negative cases. In this review, “blinded interpretation” means that the radiologists interpreting the images were not provided with any clinical history or relevant details. A study was classified as having the images interpreted by a breast radiologist if at least one breast radiologist was involved. When a study aimed to correlate ultrasound and MRI findings, only the MRI findings pertinent to this meta-analysis were extracted. Only the mean ADC was used if a study provided multiple ADC values. If more than two readers interpreted the ADC values, the lowest measurement among them was used, as a lower ADC is more indicative of malignancy [8]. Finally, when both training and validation datasets were available, the dataset with the most comprehensive information was selected. The definitions of each NME descriptor were strictly aligned with the BI-RADS guidelines [14].

We employed the Revised Tool for the Quality Assessment of Diagnostic Accuracy Studies (QUADAS-2) to evaluate the risk of bias. QUADAS-2 had no official cut off scores, and bias risk was displayed graphically [19]. Two reviewers (NK and NS) evaluated the scale independently, and any disagreements were resolved internally and through an expert decision (VNT) until a consensus was reached. We emailed the associated authors to determine if any data was missing or incomplete.

### 2.4. Data Synthesis

If the original articles did not report sensitivity, specificity, positive likelihood ratio (LR+), negative likelihood ratio (LR−), positive predictive value (PPV), or negative predictive value (NPV), these metrics were calculated manually. Sensitivity analyses were conducted to verify and account for potential outliers. A bivariate model was employed to estimate both individual and pooled sensitivity and specificity values, as recommended for diagnostic accuracy studies [20]. To illustrate the balance between sensitivity and specificity, a hierarchical summary receiver operating characteristic (HSROC) model was used, producing a summary receiver operating characteristic (SROC) curve [21]. The area under the curve (AUC) was interpreted as follows: 0.9–1.0 signified excellent accuracy, 0.8–0.9 very good, 0.7–0.8 good, 0.6–0.7 sufficient, and 0.5–0.6 poor diagnostic performance [22]. Heterogeneity across studies was quantified using the I^2^ statistic, where a value of 0% indicates no heterogeneity, and values above 50% suggest substantial heterogeneity. Cochran’s Q test was applied to determine the statistical significance of this variation [23]. To assess publication bias, a linear regression test of funnel plot asymmetry was performed, with a slope coefficient below 0.1 indicating significant bias. Fagan’s nomograms, based on Bayes’ theorem and likelihood ratios, were generated to estimate post-test probabilities. A threshold of <−0.1 for LR− and >+10 for LR+ was used to signify a meaningful change in diagnostic probability [24]. Additionally, probability modifying plots and predictive value curves were included; curves approaching the (0, 1) point reflected highly informative positive results, while those nearing (1, 0) indicated highly informative negative results [25]. Studies reporting zero values for both true positives (TP) and false negatives (FN) were excluded from meta-analysis due to computational constraints caused by infinite likelihood ratios. A regression-based meta-analysis was conducted only when ten or more studies were available for a given subgroup. The meta-analysis was carried out using the STATA program (Version 17.0, StataCorp, College Station, TX, USA) using the “midas” commands [26].

## 3. Results

**Figure 1:**
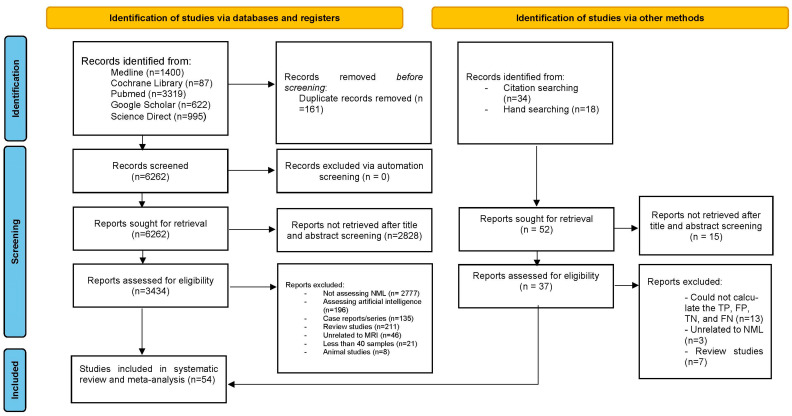
PRISMA flowchart for selection of included studies.

A total of 6423 articles were identified, with 161 duplicates removed, leaving 6262 unique articles for screening. After reviewing the titles and abstracts, 2828 articles were excluded, resulting in the final 40 studies being included in the meta-analysis [5,7,10,27,28,29,30,31,32,33,34,35,36,37,38,39,40,41,42,43,44,45,46,47,48,49,50,51,52,53,54,55,56,57,58,59,60,61,62,63]. Additional hand and citation searches identified 14 more articles [64,65,66,67,68,69,70,71,72,73,74,75,76,77]. In total, 54 articles were included in this systematic review and meta-analysis (Figure 1). Notable exclusions are presented in Supplementary Table S2.

The data reveal 4582 patients with 6121 non-mass enhancement (NME) lesions, of which 3593 are malignant, resulting in a 58.7% cancer prevalence. Among the 3054 reported malignancies, 1147 are DCIS, representing 37.6% of all malignancies. Participant age was reported in 35 studies, with an average of 49.8 (±8.4) years (Supplementary Table S3).

Variations in the number of malignant NME lesions arise because some studies do not specify the number of DCIS cases within malignant categories. Most studies (N = 37) use a retrospective design, with only 1 adopting a prospective approach. Overall, 4 studies employ a cross-sectional design, and 12 do not clarify their methodology. While 29 studies do not mention consecutive sampling, 25 explicitly report using it. Geographically, most studies were conducted in China (N = 15), Japan (N = 11), and the United States (N = 7). The three most common reasons for MRI were pre-operative staging, high-risk screening, and investigating ambiguous imaging findings (Supplementary Table S3).

**Figure 2:**
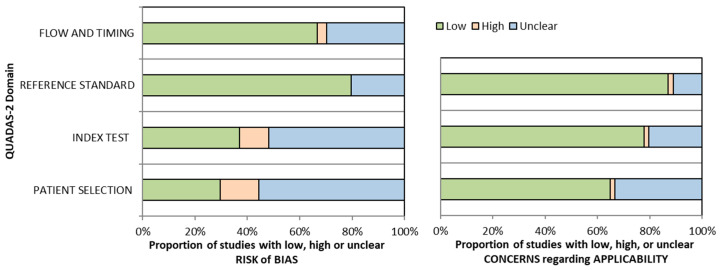
QUADAS-2 graphical representation of the risk of bias.

Most studies in this review use a 1.5T magnet strength (N = 34) with breast coils (N = 31) in the prone position (N = 42). The size of benign lesions ranges from 2 mm [75] to 92 mm [47], while malignant lesions range from 2.4 mm [50] to 150 mm [64] (Supplementary Table S4). All studies have a risk of bias or applicability concerns as they have a high or unclear risk in almost all aspects. Only two studies [66,71] have a low risk of bias (Table 1 and Figure 2).

### 3.1. Dynamic Contrast-Enhanced Magnetic Resonance Imaging

**Figure 3:**
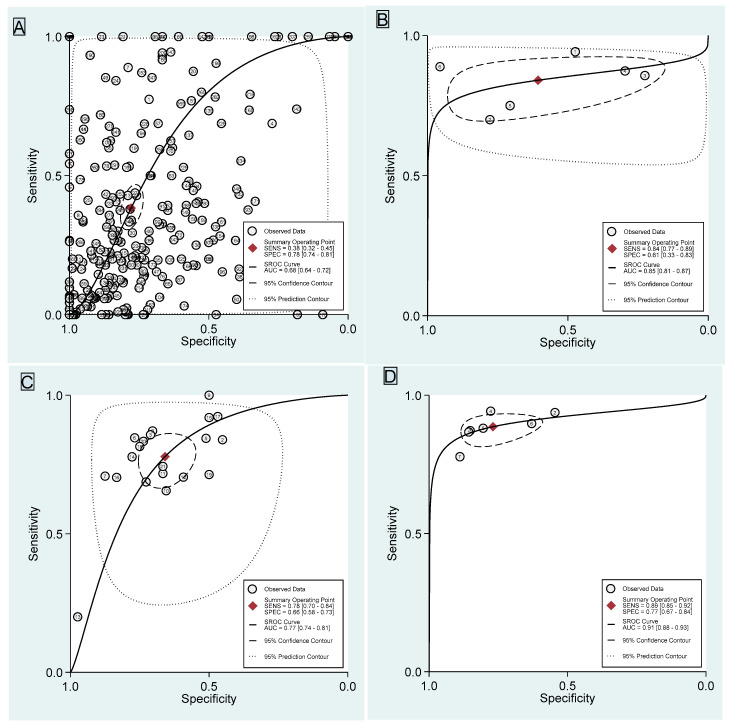
Summary receiver operating curve with confidence and prediction regions of (**A**) DCE-MRI, (**B**) DWI, (**C**) ADC, and (**D**) DCE+DWI.

**Figure 4:**
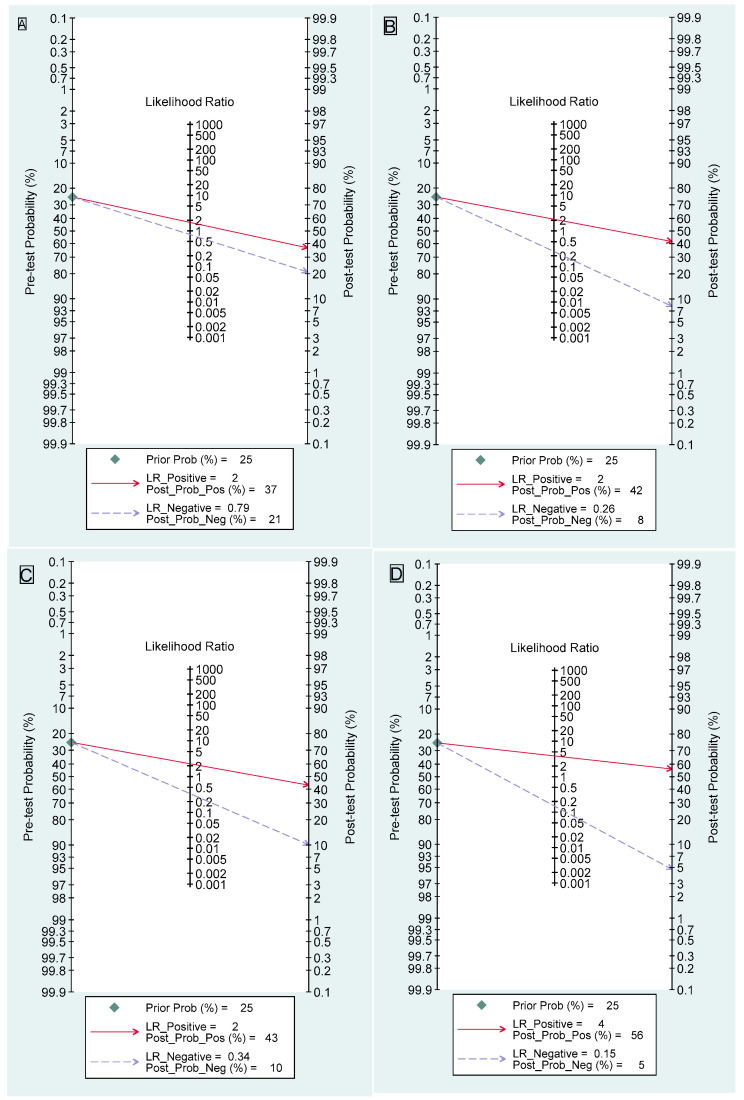
Fagan’s nomogram of (**A**) DCE-MRI, (**B**) DWI, (**C**) ADC, and (**D**) DCE+DWI.

The values obtained from DCE-MRI are derived by combining all the BI-RADS MRI descriptors, including distribution, time intensity curves, and internal enhancement characteristics. Out of all 304 observation data points, the sensitivity for DCE-MRI is 38% (95% confidence interval [CI] 32–45), and the specificity is 78% (95% CI 74–81). The combined SROC yields an AUC of 0.68 (95% CI 0.64–0.72), indicating sufficient diagnostic accuracy (Figure 3A). The I^2^ value is 100% (95% CI 100–100) with a *p* value of 0.19, indicating a non-significant detected heterogeneity. Figure 4A displays Fagan’s nomogram, showing that the LR+ is 1.73 (95% CI 1.45–2.07) with a 37% post-test probability (a 12% increase from the baseline), while the LR− is 0.79 (95% CI 0.72–0.87) with a 21% post-test probability (a 4% decrease from the baseline). The likelihood ratio scattergram placed the summary point of likelihood ratios in the right lower quadrant, meaning that DCE-MRI cannot exclude or confirm benign or malignant NME lesions (Supplementary Figure S1A). The probability modifying plot tends to the (0,1) line, indicating a more informative positive result. The combined negative predictive value is 0.55 (95% CI 0.53–0.58), and the positive predictive value is 0.62 (95% CI 0.58–0.67) (Supplementary Figure S2A). The linear regression test for funnel plot asymmetry yields a *p* value of <0.01, indicating a significant asymmetry, and thus, there is a chance of publication bias (Supplementary Figure S3A).

The meta-regression analysis showed that a 3T magnet strength significantly influenced sensitivity and specificity, with substantial heterogeneity. Using a 3T magnet increased sensitivity to 80% (95% CI: 67–88) but reduced specificity to 68% (95% CI: 57–76), with a *p* value < 0.01 and an I^2^ value of 95 (95% CI: 91–99; *p* value < 0.01). Breast coils and the number of dynamic phases only influenced sensitivity significantly, while breast radiologists and blind interpretation significantly affected the specificity (Supplementary Table S5 and Supplementary Figure S4A).

In the distribution category, segmental distribution had the highest sensitivity (65%; 95% CI 47–80%), while the multiple distribution demonstrated the highest specificity (96%; 95% CI 92–98%). Segmental distribution provided the best positive likelihood ratio of 2.95 (95% CI 2.09–4.14), whereas the multiple distribution had the best AUC at 0.89 (95% CI 0.86–0.91). Among all parameters, segmental distribution achieved the highest posterior probability of positive likelihood ratio (50%) and negative likelihood ratio (13%), assuming a baseline 25% prior probability. However, heterogeneity was substantial across all parameters. Publication bias was only apparent in the linear distribution (*p* = 0.03). The likelihood ratio scattergram indicated that no parameter could reliably exclude or confirm outcomes (Supplementary Table S6).

In the time intensity curve (TIC) category, the washout parameter demonstrated the highest specificity (87%; 95% CI 74–94%) and the best positive likelihood ratio (4.4; 95% CI 2.4–8.1). It also achieved the highest posterior probability for a positive likelihood ratio at 60% and a negative likelihood ratio at 14%, assuming a baseline of 25% prior probability. Additionally, washout had the best area under the curve (AUC) at 0.84 (95% CI 0.81–0.87). However, substantial heterogeneity was noted across all parameters, with I^2^ values of 98–99% (*p* < 0.001). Publication bias was not significant for any parameter. The likelihood ratio scattergram indicated that no parameter could reliably exclude or confirm outcomes (Supplementary Table S7).

In the internal enhancement pattern (IEP) category, clustered ring demonstrated the highest sensitivity (63%; 95% CI 38–83%) and specificity (84%; 95% CI 66–93%). It provided the best positive likelihood ratio of 3.9 (95% CI 2.2–7) and achieved the highest posterior probability of positive likelihood ratio (56%), assuming a 25% prior probability baseline. The clustered ring also had the best negative likelihood ratio (0.44; 95% CI 0.26–0.75) and the highest area under the curve (AUC) at 0.82 (95% CI 0.78–0.85). Furthermore, it exhibited the highest negative predictive value (0.68; 95% CI 0.60–0.76). Substantial heterogeneity was observed across all parameters (I^2^ > 99%; *p* < 0.001), and publication bias was not significant for clustered ring (*p* = 0.36). However, the likelihood ratio scattergram indicated that no parameter, including clustered ring, could reliably exclude or confirm outcomes (Supplementary Table S8).

### 3.2. Diffusion-Weighted Imaging

Six studies evaluate DWI [5,33,34,39,53,55] with a total of 667 lesions. The sensitivity is 84% (95% CI 77–89), and the specificity is 61% (95% CI 33–83). The combined SROC yields an AUC of 0.85 (95% CI 0.81–0.87), indicating a very good diagnostic accuracy (Figure 3B). The I^2^ value is 95% (95% CI 90–100) with a *p* value of 0.63, indicating a non-significant detected heterogeneity. Figure 4B displays Fagan’s nomogram, showing that the LR+ is 2.13 (95% CI 1.11–4.1) with a 42% post-test probability (a 17% increase from the baseline), while the LR− is 0.26 (95% CI 0.17–0.41) with an 8% post-test probability (a 17% decrease from the baseline). The likelihood ratio scattergram placed the summary point of likelihood ratios in the right lower quadrant, meaning DWI cannot exclude or confirm benign or malignant NME lesions (Supplementary Figure S1B). The probability modifying plot tends to the (1,0) line, indicating a more informative negative result. The combined negative predictive value is 0.77 (95% CI 0.63–0.92), and the positive predictive value is 0.67 (95% CI 0.55–0.78) (Supplementary Figure S2B). The linear regression test for funnel plot asymmetry yields a *p* value of 0.05, indicating a significant asymmetry, and thus, there is a chance of publication bias (Supplementary Figure S3B). No meta-regression can be performed since there are fewer than 10 studies.

### 3.3. Apparent Diffusion Coefficient

Twenty-one studies evaluate ADC [5,7,10,27,28,33,36,37,38,40,41,44,45,46,47,51,52,58,62,66,77] with a total of 1858 lesions. The specific cut off used from each study is available in Supplementary Table S9. One study [54] is excluded as both FP and TN are 0. The sensitivity is 78% (95% CI 70–84), and the specificity is 66% (95% CI 58–73). The combined SROC yields an AUC of 0.77 (95% CI 0.74–0.81), indicating a good diagnostic accuracy (Figure 3C). The I^2^ value is 95% (95% CI 90–99) with a *p* value of <0.0001, indicating a significantly detected heterogeneity. Figure 4C displays Fagan’s nomogram, showing that the LR+ is 2.28 (95% CI 1.81–2.87) with a 43% post-test probability (an 18% increase from the baseline), while the LR− is 0.34 (95% CI 0.24–0.47) with a 10% post-test probability (a 15% decrease from the baseline). The likelihood ratio scattergram placed the summary point of likelihood ratios in the right lower quadrant, meaning that ADC cannot exclude or confirm benign or malignant NME lesions (Supplementary Figure S1C). The probability modifying plot tends to the (1,0) line, indicating a more informative negative result. The combined negative predictive value is 0.73 (95% CI 0.68–0.78), and the positive predictive value is 0.68 (95% CI 0.63–0.73) (Supplementary Figure S2C). The linear regression test for funnel plot asymmetry yields a *p* value of 0.88, indicating a non-significant asymmetry, and thus, there is a low chance of publication bias (Supplementary Figure S3C).

Meta-regression is performed on 20 studies, as 1 study failed to provide the ADC cut off [45]. Only axial scanning significantly affects the specificity, with an increase of 82% (95% CI 69–90) and a *p* value of 0.03 (Supplementary Table S10 and Supplementary Figure S4B). The b value variable was not included as all studies used high b values of 800 s/mm^2^.

### 3.4. Dynamic Contrast-Enhanced Magnetic Resonance Imaging Combined with Diffusion-Weighted Imaging

Seven studies evaluate DCE+DWI [5,27,28,37,39,41,52] with a total of 649 lesions. The sensitivity is 89% (95% CI 85–92), and the specificity is 77% (95% CI 67–84). The combined SROC yields an AUC of 0.91 (95% CI 0.88–0.93), indicating an excellent accuracy (Figure 3D). The I^2^ value is 10% (95% CI 0–100) with a *p* value of 0.164, indicating a low probability of heterogeneity, although the finding is insignificant. Figure 4D displays Fagan’s nomogram, showing that the LR+ is 3.84 (95% CI 2.67–5.52) with a 56% post-test probability (a 31% increase from the baseline), while the LR− is 0.15 (95% CI 0.11–0.20) with a 5% post-test probability (a 20% decrease from the baseline). The likelihood ratio scattergram placed the summary point of likelihood ratios in the right lower quadrant, meaning that DCE+DWI cannot be used to exclude or confirm benign or malignant NME lesions (Supplementary Figure S1D). The probability modifying plot tends to the (1,0) line, indicating a more informative negative result. The combined negative predictive value is 0.86 (95% CI 0.80–0.91), and the positive predictive value is 0.78 (95% CI 0.72–0.83) (Supplementary Figure S2D). The linear regression test for funnel plot asymmetry yields a *p* value of 0.45, indicating a non-significant asymmetry, and thus, there is a low chance of publication bias (Supplementary Figure S3D). No meta-regression can be performed since there are less than 10 studies.

## 4. Discussion

This meta-analysis shows that the combination of DCE-MRI and DWI had the highest performance with an AUC of 0.91. DWI alone showed a very good AUC of 0.85, while ADC values achieved a good AUC of 0.77. DCE-MRI shows the poorest AUC of 0.68. Based on the likelihood ratio scattergram, these four modalities could neither confirm nor exclude malignancies. Among the descriptors, the washout TIC parameter, the clustered ring IEP, and the segmental distribution have the best likelihood ratios in predicting malignancies. However, these descriptors also could neither confirm nor exclude malignancies. This meta-analysis included 54 studies, more than four times the amount included in the previous meta-analyses [11,12].

DCE-MRI demonstrates the lowest diagnostic accuracy among the modalities examined. The pooled sensitivity and specificity for DCE-MRI in our analysis, 38%, and 78%, are comparable to previously reported values of 50% and 80% [11] as well as 58% and 72% [12], respectively. One key factor contributing to this is the variability in the accuracy of many descriptors, making the sole reliance on DCE-MRI less reliable [78]. The interobserver variability for NME descriptors shows only low to moderate agreement. While studies suggest this variability is statistically significant, it does not substantially affect diagnostic performance [60,79]. Our meta-regression shows that when the results were interpreted solely based on breast radiologists, the specificity dropped to only 67%.

Another potential reason is the heterogeneous mix of benign and malignant lesions in the studies, as the prevalence of cancers may affect diagnostic test accuracy [11]. Although most NME malignancies are DCIS, other types, such as invasive ductal carcinoma and invasive lobular carcinoma, are also represented. Benign NME lesions vary widely and include conditions such as pseudoangiomatous stromal hyperplasia, apocrine metaplasia, and radiation-induced changes. Furthermore, high-risk or “mixed category” lesions complicate accuracy assessments. Depending on the study, these lesions may be considered benign or malignant, including atypical ductal hyperplasia, radial scars, complex sclerosing lesions, flat epithelial atypia, and intraductal papillomas. Compounding these challenges is the significant overlap in imaging features between benign, high-risk, and malignant processes in the breast, further affecting the diagnostic precision of DCE-MRI [80].

Segmental distribution [60], clustered ring enhancement patterns [81], and washout (type III) curves [82] are commonly associated with malignancies in NME, as supported by this meta-analysis. These features alone cannot reliably confirm or exclude malignancy. Similarly, the “multiple regions” descriptor, while unable to definitively confirm or rule out malignancies, demonstrates the second-highest diagnostic accuracy among the analyzed patterns—a finding that has received limited attention in previous studies. One possible explanation is that individual studies often include only a small number of lesions exhibiting the “multiple regions” descriptor, limiting further analysis. However, through pooled meta-analysis, the potential diagnostic value of this descriptor becomes evident.

Contrast-enhanced breast MRI findings are classified as “multiple” when at least two large volumes of tissue are involved, separated by normal breast tissue or fat. While diffuse and multiple regions of contrast enhancement are generally associated with benign proliferative changes, such as mammary gland hyperplasia, multicentric breast carcinoma can also exhibit multiple distributions, highlighting the importance of careful interpretation [70,80,83].

Diffusion-weighted imaging is a valuable tool for distinguishing between benign and malignant breast lesions, stratifying in situ from invasive disease, and potentially predicting and monitoring the response to neoadjuvant treatment over time [8]. DWI provides quantitative information essential for lesion characterization by being sensitive to tissue microstructure and cellularity. This enhanced characterization can help reduce unnecessary biopsy recommendations [51]. The quantitative nature of ADC measurements, combined with relatively short acquisition times of 2–4 min (not exceeding 5 min), positions DWI as an ideal candidate for an imaging biomarker [84]. The ADC values in breast mass lesions are reliable for distinguishing between benign and malignant cases [85].

However, current challenges include significant variability in reported specificity, sensitivity, and ADC thresholds for distinguishing between benign and malignant breast lesions. Another critical issue is inconsistent image quality due to variations in MRI system capabilities, equipment, and imaging protocols, contributing to a perceived limitation of DWI in clinical practice. These challenges and the absence of prospectively validated thresholds to guide diagnostic decisions have hindered the integration of DWI into the BI-RADS [8]. Progress has been made toward standardizing protocols, with most studies now employing at least a 1.5T MRI equipped with dedicated breast coils and conducting scans in the prone position. Additionally, nearly all studies have adopted a b value of 800 s/mm^2^ or higher, regarded as optimal for imaging [8,86].

Significant heterogeneities remain in sequences, imaging orientations, field-of-view, in-plane resolution, slice thickness, number of b values, echo time (TE), repetition time (TR), and post-processing methods used across institutions and centers. Furthermore, variations in how the region of interest (ROI) is selected and interpreted can influence results [86]. Several studies do not consistently report their ADC values, as some report only the minimum, maximum, or even the mean values [8]. Additionally, whether quality control measures are implemented before scanning impacts the inter-scan reproducibility of diffusion index measurements [87]. This meta-analysis could not assess these factors due to insufficient or highly heterogeneous data, making meta-regression unfeasible.

The European Society of Breast Radiology (EUSOBI) categorizes ADC values into five ranges: very low (≤0.9 × 10^−3^ mm^2^/s), low (0.9–1.3 × 10^−3^ mm^2^/s), intermediate (1.3–1.7 × 10^−3^ mm^2^/s), high or normal (1.7–2.1 × 10^−3^ mm^2^/s), and very high (>2.1 × 10^−3^ mm^2^/s) [8]. A previous meta-analysis reported that an ADC cut off of <1.3 × 10^−3^ mm^2^/s had slightly higher sensitivity (86%) than a cut off of ≥1.3 × 10^−3^ mm^2^/s with 82%. Still, both cut offs showed limited specificity (67% and 68%, respectively) [12]. Our meta-analysis suggests that a stricter cut off of <1 × 10^−3^ mm^2^/s reduces sensitivity to 80% but improves specificity to 75%. Increasing the cut off to <1.5 × 10^−3^ mm^2^/s raises specificity to 81%, though sensitivity drops to 64%. Thus, lower diffusion cut offs favor sensitivity at the expense of specificity, while intermediate cut offs enhance specificity at the cost of sensitivity. Given these trade offs, and until more robust results are available, the authors concur with EUSOBI’s recommendation against providing specific quantitative cut off values [12].

The combination of DCE-MRI and DWI, commonly called multiparametric MRI (Mp-MRI), demonstrates the highest diagnostic accuracy, consistent with the findings from previous meta-analyses [12]. This approach aims to enhance specificity and reduce false-positive results, as our findings concur with this statement. In a meta-analysis focused on breast mass lesions, Mp-MRI did not improve sensitivity but significantly increased specificity for diagnosing malignant breast lesions [88].

Although combining DCE-MRI with DWI appears promising for differentiating benign and malignant lesions in NME, challenges such as time constraints in clinical practice and limited clinical utility—evidenced by low LR+, LR−, PPV, and NPV—underscore the need for more robust studies [37,89]. These additional investigations are essential before considering its inclusion in BI-RADS guidelines.

Despite our best efforts to conduct a meta-regression analysis, several other parameters may still contribute to the observed heterogeneity. First, the studied population may act as a confounding factor. In screening populations, NME typically has a lower malignancy rate compared with NMEs detected during staging or diagnostic workup for a known lesion. For instance, one study reported a PPV of 14% and 10% when MRI was perfosrmed for screening purposes [90]. In contrast, NMEs identified during staging are often associated with a higher likelihood of malignancy, particularly when accompanied by imaging features suggestive of disease spread [91]. Unfortunately, many studies do not differentiate between these populations, making meta-regression analysis to explore this effect infeasible. Second, the proportion of DCIS varied widely across the included studies, ranging from 0% to 95.9%. Similar to the population factor, studies with a higher proportion of DCIS may involve more invasive disease processes, further contributing to heterogeneity. Third, heterogeneity in imaging protocols across institutions leads to variability in reported sensitivities and specificities, which cannot be fully accounted for in the meta-regression. For example, one study demonstrated that combining DCE-MRI with DWI and turbo inversion recovery magnitude (TIRM) sequences improved diagnostic performance in differentiating NME lesions compared with DCE-MRI alone [41]. However, not all studies incorporated the TIRM sequence, potentially influencing the diagnostic outcomes. Lastly, the differences in study design may also affect the results. Cohort studies generally offer more robust data than cross-sectional studies, though a randomized controlled trial may ultimately be required to validate these findings.

This study has several inherent limitations. First, heterogeneity is evident across all modalities examined, and meta-regression could not identify its underlying causes. In addition to the previously discussed factors, other plausible contributors include the diverse range of patient presentations and the varying purposes of MRI. For example, the results differ between women undergoing MRI for pre-operative staging and those undergoing MRI for dense breast screening. Although magnet strength and breast coil type emerged as important factors influencing sensitivity in the meta-regression analysis for DCE-MRI, considerable heterogeneity remains. These parameters were not significantly associated with the ADC values. Consequently, prospective studies using standardized protocols and homogenous patient samples are warranted to determine the optimal MRI acquisition parameters. As this issue lies beyond the scope of our meta-analysis, we were unable to establish definitive recommendations regarding MRI protocols, including magnet strength, coil configuration, or patient positioning. Furthermore, some studies included a broad range of BI-RADS categories (3 to 6), while others specifically targeted BI-RADS 4. Second, evaluating combinations of BI-RADS descriptors was not feasible due to the lack of available data. While individual descriptors, such as segmental distribution, clustered ring enhancement patterns, and washout curves, cannot independently confirm or exclude malignancies, a combination of two or all three may represent a critical diagnostic marker for NME malignancies. However, that is beyond the scope of this meta-analysis. Third, only two studies included in the meta-analysis were identified as having a low risk of bias, meaning that the results are inherently influenced by studies with an intermediate to high risk of bias. According to Cochrane guidelines, studies with a high risk of bias should not be excluded but assessed through subgroup analysis [92]. However, since most studies included some bias, a subgroup analysis would likely yield similar results. Fourth, some BI-RADS descriptors, such as background parenchymal enhancement and the initial upslope of kinetic curve assessment, could not be assessed as not all studies included these data. Finally, publication bias was noted for both DCE-MRI and DWI, likely due to the “file-drawer” effect, where studies with favorable outcomes are more likely to be published [93].

Despite its limitations, this meta-analysis represents the most comprehensive and systematic review conducted on this topic to date, to the best of the authors’ knowledge. The findings indicate that DCE-MRI, DWI, ADC, and the combination of DCE-MRI and DWI are not clinically sufficient for reliably differentiating benign from malignant lesions, contrary to the conclusions of other meta-analyses [11,12]. Additionally, this meta-analysis suggests that the “multiple regions” descriptor shows potential and warrants further investigation in future studies.

## 5. Conclusions

This comprehensive meta-analysis underscores that while individual diagnostic modalities like DCE-MRI, DWI, and ADC demonstrate varying diagnostic accuracy, their limitations preclude their standalone clinical reliability for differentiating benign from malignant NME. Although promising, with the highest diagnostic accuracy among the examined techniques, the combined use of DCE-MRI and DWI also falls short of providing definitive diagnostic certainty. Key imaging descriptors such as segmental distribution, clustered ring, and washout patterns show potential utility but remain insufficient in isolation.

The findings emphasize the complexity of diagnosing NME, reflecting significant heterogeneity across studies, imaging protocols, and lesion characteristics. These results advocate for a cautious approach to integrating these modalities into clinical practice and call for further research to refine imaging criteria, standardize protocols, and explore the potential of combined diagnostic descriptors. Achieving greater accuracy will require multidisciplinary efforts to improve diagnostic technologies and methodologies. With the upcoming BI-RADS edition, it will be intriguing to observe how the descriptors for NME evolve. Future research should focus on integrating advanced imaging technologies, such as radiomics and machine learning, to develop predictive models that improve diagnostic confidence. Additionally, large-scale, prospective multicenter studies are needed to validate combined imaging descriptors and establish standardized thresholds for clinical application.

## Figures and Tables

**Table 1:** QUADAS-2 result of each study.

Study	Risk of Bias				Applicability Concerns			Conclusions
	Patient Selection	Index Test	Reference Standard	Flow and Timing	Patient Selection	Index Test	Reference Standard	
Yabuuchi (2009) [27]	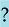	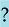	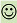	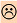	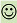	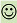	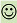	High risk of bias
Baltzer (2011) [75]	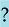	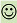	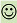	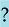	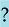	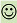	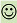	At risk of bias
Sakamoto (2008) [29]	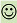	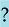	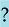	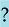	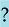	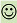	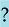	At risk of bias
Imamura (2010) [28]	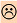	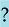	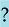	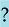	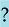	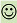	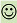	High risk of bias
Sotome (2007) [30]	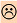	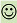	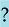	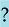	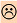	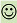	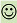	High risk of bias
Baltzer (2010) [76]	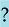	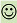	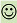	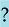	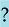	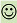	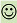	At risk of bias
Goto (2007) [74]	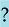	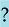	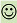	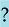	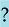	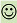	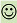	At risk of bias
Thomassin-Naggara (2011) [31]	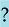	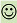	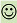	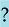	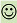	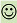	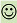	At risk of bias
Tozaki (2005) [67]	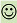	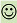	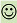	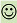	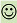	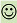	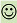	Low risk of bias
Tokuda (2009) [73]	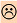	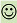	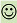	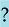	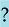	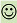	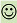	High risk of bias
Yuen (2008) [72]	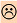	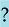	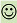	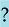	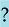	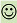	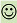	High risk of bias
Liberman (2002) [59]	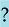	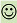	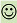	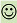	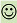	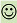	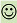	At risk of bias
Liberman (2003) [71]	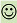	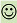	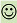	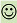	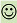	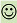	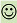	Low risk of bias
Di Ninno (2021) [32]	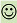	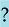	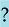	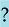	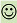	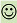	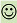	At risk of bias
Moukhtar (2014) [77]	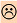	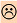	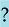	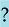	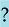	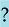	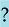	High risk of bias
Liu (2022) [33]	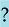	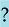	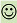	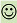	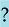	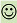	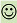	At risk of bias
Aydin (2019) [34]	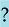	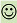	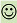	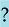	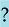	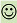	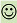	At risk of bias
Zhou (2021) [35]	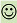	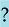	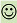	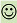	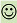	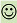	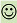	At risk of bias
Lunkiewicz (2020) [60]	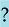	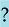	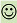	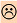	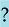	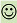	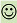	High risk of bias
Uematsu & Kasami (2012) [64]	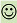	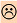	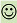	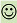	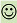	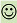	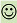	High risk of bias
Chikarmane (2017) [70]	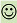	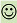	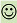	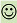	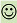	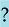	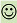	At risk of bias
Cheng (2013) [36]	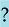	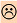	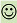	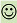	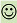	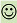	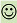	High risk of bias
Marino (2022) [37]	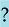	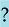	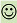	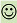	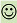	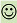	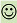	At risk of bias
Kul (2013) [38]	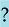	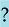	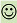	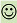	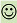	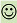	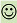	At risk of bias
Yang (2020) [39]	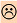	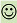	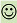	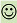	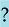	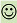	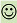	High risk of bias
Liu (2023) [40]	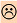	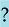	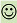	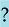	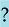	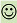	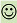	High risk of bias
Zang (2022) [41]	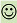	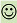	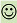	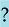	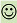	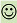	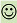	At risk of bias
Bilge (2022) [42]	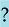	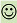	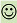	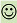	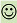	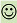	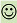	At risk of bias
Kwon (2020) [43]	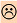	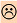	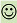	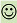	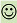	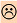	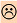	High risk of bias
Liu (2020) [56]	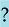	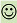	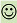	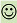	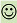	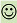	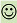	At risk of bias
Lv (2022) [44]	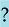	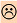	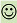	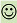	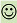	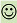	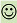	High risk of bias
Zhao (2020) [47]	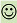	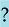	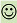	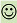	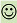	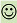	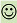	At risk of bias
Zhang (2022) [45]	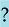	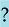	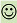	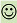	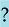	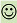	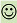	At risk of bias
Li (2023) [46]	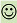	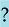	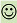	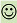	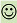	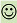	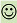	At risk of bias
Yang (2017) [48]	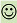	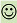	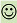	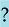	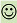	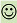	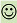	At risk of bias
Chen (2021) [49]	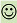	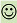	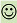	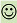	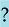	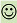	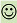	At risk of bias
Ballesio (2014) [65]	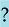	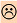	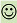	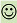	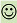	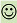	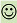	High risk of bias
Avendano (2019) [7]	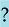	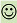	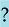	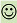	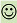	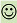	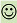	At risk of bias
Asada (2017) [50]	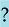	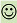	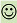	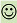	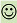	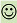	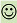	At risk of bias
Bayoumi (2024) [5]	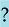	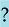	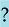	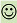	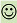	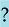	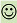	At risk of bias
Spick (2014) [51]	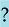	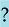	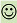	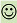	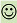	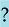	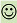	At risk of bias
Tang (2021) [58]	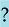	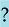	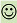	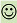	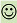	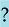	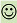	At risk of bias
Clauser (2021) [62]	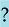	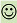	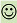	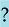	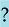	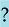	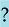	At risk of bias
Jansen (2011) [57]	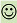	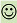	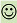	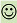	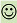	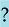	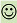	At risk of bias
Li (2023) [52]	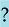	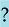	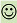	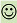	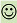	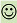	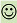	At risk of bias
Cho (2016) [63]	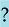	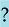	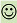	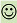	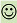	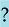	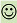	At risk of bias
Partridge (2010) [10]	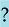	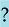	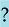	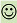	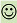	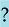	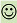	At risk of bias
Niu (2023) [54]	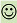	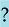	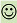	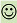	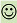	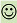	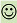	At risk of bias
Mohamed (2024) [53]	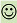	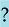	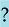	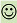	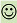	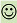	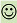	At risk of bias
Ahmadinejad (2024) [55]	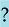	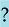	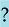	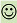	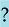	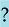	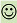	At risk of bias
Kim (2021) [61]	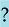	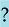	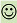	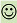	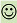	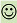	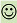	At risk of bias
Tozaki & Fukuma (2009) [66]	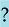	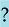	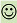	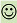	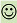	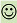	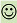	At risk of bias
Gity (2014) [69]	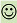	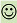	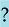	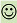	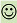	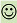	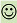	At risk of bias
Wilhelm (2012) [68]	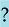	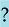	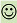	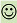	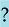	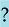	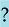	At risk of bias

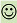
 Low Risk; 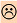 High Risk; 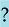 Unclear Risk.

## Data Availability

All available data are presented in the Supplementary Material.

## Supplementary Materials

The following supporting information can be downloaded at: https://www.mdpi.com/article/10.3390/jcm14134628/s1, Figure S1: Meta-regression of DCE-MRI (A) and ADC (B); Figure S2: Likelihood ratio scattergram of (A) DCE-MRI, (B) DWI, (C) ADC, and (D) DCE+DWI; Figure S3: Probability modifying plot of (A) DCE-MRI, (B) DWI, (C) ADC, and (D) DCE+DWI; Figure S4: Linear regression test of funnel plot asymmetry of (A) DCE-MRI, (B) DWI, (C) ADC, and (D) DCE+DWI; Table S1: Medical subject heading (MeSH) terms used in each database; Table S2: Notable exclusions; Table S3: Descriptive findings of each study, Table S4: Diagnostic test parameters of included studies; Table S5: Meta-regression analysis of DCE-MRI; Table S6: Pooled estimates of diagnostic performance of dynamic contrast enhancement of distribution pattern; Table S7: Pooled estimates of diagnostic performance of dynamic contrast enhancement of time intensity curve pattern; Table S8: Pooled estimates of diagnostic performance of dynamic contrast enhancement of internal enhancement pattern; Table S9: ADC cut off from each study; Table S10: Meta-regression analysis of ADC.

## Abbreviations

The following abbreviations are used in this manuscript:

ADC	Apparent Diffusion Coefficient
AUC	Area Under the Curve
BI-RADS	Breast Imaging Reporting and Data System
CI	Confidence Interval
DCIS	Ductal Carcinoma In Situ
DCE-MRI	Dynamic Contrast-Enhanced Magnetic Resonance Imaging
DWI	Diffusion-Weighted Imaging
FN	False Negative
FP	False Positive
HSROC	Hierarchical Summary Receiver Operating Characteristic
IEP	Internal Enhancement Pattern
LR+	Positive Likelihood Ratio
LR−	Negative Likelihood Ratio
Mp-MRI	Multiparametric Magnetic Resonance Imaging
MRI	Magnetic Resonance Imaging
NME	Non-Mass Enhancement
NPV	Negative Predictive Value
PPV	Positive Predictive Value
PRISMA-DTA	Preferred Reporting Items for Systematic Review and Meta-Analysis of Diagnostic Test Accuracy
PROSPERO	International Prospective Register of Systematic Reviews
QUADAS-2	Quality Assessment of Diagnostic Accuracy Studies-2
ROI	Region of Interest
SROC	Summary Receiver Operating Characteristic
TE	Echo Time
TN	True Negative
TP	True Positive
TR	Repetition Time
