# Supplementary material for: An Updated Systematic Review and Meta-Analysis of Diagnostic Accuracy of Dynamic Contrast Enhancement and Diffusion-Weighted MRI in Differentiating Benign and Malignant Non-Mass Enhancement Lesions
Source: J Clin Med. 2025 Jun 30;14(13):4628. doi: 10.3390/jcm14134628 (PMC12250181; doi:10.3390/jcm14134628)
Supplement: Supplementary file 1 [file jcm-14-04628-s001.zip › jcm-3678019-supplementary.pdf]

Journal of
Clinical Medicine

MDPI

Supplementary Table S1. Medical subject heading (MeSH) terms used in each database.

Database	Medical Subject Heading	Number of studies found
Pubmed	("breast"[MeSH Terms] OR "breast"[All Fields] OR "breasts"[All Fields] OR "breast s"[All Fields]) AND (("Non"[All Fields] AND ("molecular weight"[MeSH Terms] OR ("molecular"[All Fields] AND "weight"[All Fields]) OR "molecular weight"[All Fields] OR "mass"[All Fields])) OR "non-mass-like"[All Fields] OR ("non-mass"[All Fields] AND ("enhance"[All Fields] OR "enhanced"[All Fields] OR "enhancement"[All Fields] OR "enhancements"[All Fields] OR "enhancer"[All Fields] OR "enhancer s"[All Fields] OR "enhancers"[All Fields] OR "enhances"[All Fields] OR "enhancing"[All Fields])) OR ("non-mass"[All Fields] AND ("lesion"[All Fields] OR "lesion s"[All Fields] OR "lesional"[All Fields] OR "lesions"[All Fields]))) AND ("magnetic resonance imaging"[MeSH Terms] OR ("magnetic"[All Fields] AND "resonance"[All Fields] AND "imaging"[All Fields]) OR "magnetic resonance imaging"[All Fields] OR ("diagnostic imaging"[MeSH Subheading] OR ("diagnostic"[All Fields] AND "imaging"[All Fields]) OR "diagnostic imaging"[All Fields]))	3319
Medline	((((((non-mass lesion) OR non-mass enhancement) OR non-mass-like) OR non-mass)) AND (((magnetic resonance imaging) OR AND (breast)	1400
Cochrane Library	(breast non-mass lesion):ti,ab,kw AND (magnetic resonance imaging):ti,ab,kw	87
Google Scholar	"breast non-mass"	398
	"breast non mass lesions"	224
Science Direct	breast non mass lesion AND (Magnetic Resonance Imaging) [Research articles only]	995

Supplementary Table S2. Notable exclusions.

Author (Year)	Reason for exclusion
Pinker (2013)[1]	There is no data separation between mass and NME lesions
El Khouli (2010)[2]	
Shimauchi (2015)[3]	
Santamaría (2013)[4]	The aim is to assess the correlation between microvascular patterns and MRI enhancement
Lee (2018)[5]	The aim is to assess the pattern of invasiveness. However, whether the lesion is malignant or not was not confirmed.
Machida (2016)[6]	
Morakkabati-Spitz (2005)[7]	Does not specify whether all lesions are NME lesions
Parsian (2012)[8]	The aim is to determine the ADC cut-off value for high-risk and benign lesions.

NME, Non-mass enhancing; MRI, Magnetic resonance imaging; ADC, Apparent diffusion coefficient.

Supplementary Table S3. Descriptive findings of each study.

Author (Year)	Design	Sampling	Country	Age (years)	Indications for MRI	Number of centre(s)	Number of radiologists	Breast radiologists	Number of patients with NME	Number of NME lesions (Number of malignant NME lesions)	Cancer prevalence (%)	Percentage of DCIS in cancer (%)	MRI diagnostic criteria	Reference standard	Blinded Interpretation

Yabuuchi (2009)[9]	Not clear	Consecutive	Japan	Mean: 55.4	Pre-operative staging, equivocal mammographic findings, palpable breast mass but uncertain mammography or sonography, nipple discharge	1	2	No	41	45 (31)	68.9	Unknown	Distribution Or IEP*	Histopathology	No
Baltzer (2011)[10]	Not clear	Consecutive	Germany	Mean: 55 (12)	Presence of biopsy-proven lesions, referral of the patient to the radiology department from the department of gynecology and obstetrics, and all lesions described as suspicious in the initial breast MRI report	1	2	No	316	65 (34)	52.3	52.9 (18/34)	Distribution Or IEP*	Histopathology	Yes
Sakamoto (2008)[11]	Retrospective	Consecutive	Japan	Mean: 39	Further work-up in 59 cases (58%); screening in 23 cases (23%); follow-up for another lesion after biopsy in 11 cases (11%); pre-operative assessment of the extent of disease in 5 cases (5%); and four symptomatic lesions (nipple discharge) (4%)	1	1	Yes	Unknown for NML	102 (10)	9.8	90 (9/10)	Distribution Or IEP*	US-VAB and/or follow-up	Yes
Imamura (2010)[12]	Not clear	Not clear	Japan	Median: 50 (30-81)	Further examinations of palpable mass lesions, bloody	1	2	Yes	27	27 (16)	59.3	18.8 (3/16)	Distribution Or IEP*	Histopathology and/or	Yes

					secretions, or abnormalities on mammography									follow-up	
Sotome (2007)[13]	Not clear	Not clear	Japan	Unknown for NML	Not mentioned	1	Not mentioned	Not mentioned	Unknown for NML	32 (18)	56.3	16.7 (3/18)	Distribution	Histopathology and/or cytology	Yes
Baltzer (2010)[14]	Not clear	Consecutive	Germany	Unknown for NML	Unclear (n = 16) or suspicious (n = 58) mammographic findings, screening because of previous breast cancer (n = 37), suspicious ultrasound findings (n = 24), follow-up of probably benign lesions (n = 9), contralateral findings (n = 5), and suspicious findings at galactography (n = 1).	1	2	No	Unknown for NML	29 (15)	51.7	13.3 (2/15)	Distribution Or IEP*	Histopathology	Yes
Goto (2007)[15]	Not clear	Consecutive	Japan	Unknown for NML	To achieve a more accurate diagnosis of breast lesions and assess the extent of a tumor before planning breast-conserving surgery.	1	2	Yes	Unknown for NML	37 (18)	49	33 (6/18)	Distribution	Histopathology and/or follow-up	Yes
Thomassin-Naggara (2011)[16]	Retrospective	Not clear	Canada	Mean: 51.4	High-risk screening, breast cancer staging, inconclusive mammography and/or sonography,	1	2	No	Unknown for NML	131 (56)	42.7	55.4 (31/56)	Distribution Or IEP*	Histopathology	Yes

					short-term follow-up, and search for an occult primary tumor and positive margin after surgery									
Tozaki (2005)[17]	Retrospective	Consecutive	Japan	Mean: 48	Not mentioned	1	2	Yes	Unknown for NML	61 (35)	57.4	85.7 (30/35)	Distribution Or IEP**	Histopathology	Yes
Tokuda (2009)(18)	Not clear	Not clear	Japan	Mean: 49	Identify lesions causing suspicious nipple discharge and determine their extent.	1	3	No	Unknown for NML	39 (17)	43.6	47.1 (8/17)	Distribution and/or IEP**	Histopathology	Yes
Yuen (2008)[19]	Retrospective	Not clear	Japan	Mean: 50.7 (13.1)	Assess the nature and extent of lesions detected by clinical examination, mammography, and/or ultrasonography	1	2	No	Unknown for NML	70 (57)	81.4	43.9 (25/57)	Distribution Or IEP**	Histopathology and/or follow-up	Yes
Liberman (2002)[20]	Retrospective	Not clear	USA	Unknown for NML	Screening of 41 lesions considered high-risk (due to a history of breast cancer, a biopsy-confirmed diagnosis of atypical ductal hyperplasia or lobular carcinoma, or a family history of breast cancer), evaluation of disease extent in 38 lesions among patients with diagnosed synchronous	1	1	No	Unknown for NML	40 (10)	25	90 (9/10)	Distribution Or IEP*	Histopathology	Yes

					cancer and resolving issues in 21 lesions (including uncertain findings from previous imaging studies or physical exams, or other issues like an occult carcinoma in an axillary lymph node suggestive of a breast primary).										
Liberman (2003)[21]	Retrospective	Not clear	USA	Median: 51 (33-79)	Screening examination for patients at high risk for breast cancer, assessment of the extent of disease in patients with known synchronous cancer, problem-solving, and follow-up for findings on a prior MR imaging study	1	1	Yes	80	88 (40)	45.5	45 (18/40)	IEP*	Histopathology and/or follow-up	Yes
Di Ninno (2021)[22]	Cross-sectional	Consecutive	Brazil	Unknown for NML	All patients who had BI-RADS 4 or 5 calcifications and who had a biopsy indication	2	3	Yes	Unknown for NML	48 (38)	79.2	44.7 (17/38)	Distribution Or IEP*	Histopathology and/or follow-up	Yes
Moukhtar (2014)[23]	Not clear	Not clear	Egypt	Unknown for NML	Not mentioned	1	1	No	Unknown for NML	39 (27)	69.2	77.8 (21/27)	ADC threshold	Histopathology	Not mentioned
Liu (2022)[24]	Retrospective	Not clear	China	18-70 years old	Not mentioned	1	2	No	118	56 (118)	47.5	27.1 (32/118)	IEP; distribution;	Histopathology	Yes

													TIC; ADC threshold		
Aydin (2019)[25]	Retrospective	Not clear	Turkey	Mean: 45.9 (11.4)	Screening of females at high risk of breast cancer, pre-operative staging of newly diagnosed breast cancer (ipsilateral and contralateral), evaluation of the effect of neoadjuvant chemotherapy, evaluation of females with breast implants, occult primary breast carcinoma, suspected local recurrence, and problem-solving	1	1	No	127	30 (129)	23.3	Not mentioned	IEP; distribution; diffusion restriction; TIC; IEP and distribution	Histopathology and/or follow-up	Not mentioned
Zhou (2021)[26]	Retrospective	Consecutive	China	Malignant: 49 (11) and benign: 45 (12)	Not mentioned	1	3	No	150	150 (104)	69.3	42.3 (44/104)	IEP; distribution; BI-RADS; IEP and distribution	Histopathology	Yes
Lunkiewicz (2020)[27]	Retrospective	Not clear	Switzerland	Mean: 52.6 (20.7)	MRI-guided vacuum-assisted core breast biopsy	2	2	Yes	72	72 (19)	26.4	31.6 (6/19)	Distribution and/or IEP**	Histopathology	Yes
Uematsu & Kasami (2012)[28]	Prospective	Consecutive	Japan	Not mentioned	Not mentioned	1	1	No	122	124 (85)	69	48 (41/85)	Distribution Or IEP*	Histopathology	No

Chikarmane (2017)[29]	Retrospective	Consecutive	USA	Mean: 48.8 (21-84)	Not mentioned	1	2	No	144	205 (52)	36.1	53.8 (28/52)	Distribution and/or IEP**	Histopathology	Yes
Cheng (2013)[30]	Not clear	Consecutive	China	Unknown for NML	Not mentioned	1	2	No	61	61 (42)	66.1	33.3 (14/42)	IEP	Histopathology	Not mentioned
Marino (2022)[31]	Retrospective	Not clear	USA	Mean: 51.8 (10.8)	Not mentioned	1	2	Yes	66	66 (39)	59	10.3 (4/39)	Distribution and/or IEP**	Histopathology and surgery	Yes
Kul (2013)[32]	Retrospective	Consecutive	Turkey	Unknown for NML	Pre-operative local staging (64%), clarification of suspicious findings from conventional imaging or clinical exams (18%), screening of high-risk women (10%), detecting residual cancer in the early postoperative phase (5%), follow-up after breast-conserving surgery (2%), and identifying occult primary breast cancer (1%)	1	1	No	Unknown for NML	73 (28)	38.4	Unknown	ADC threshold	Histopathology and/or follow-up	Yes
Yang (2020)[33]	Not clear	Not clear	China	Unknown for NML	Not mentioned	1	2	No	Unknown for NML	71 (52)	73.2	Unknown	Distribution Or IEP**	Histopathology	Yes
Liu (2023)[34]	Not clear	Not clear	China	49.67 ± 11.12	Not mentioned	1	2	No	Unknown	122 (77)	63.1	37.7 (29/77)	ADC, distribution, and/or IEP**	Histopathology	Not mentioned

Zang (2022)[35]	Retrospective	Consecutive	China	Median = 46 (28-79)	Not mentioned	1	2	Yes	193	199 (117)	58.8	17.9 (21/117)	Distribution and/or IEP**	Histopathology	Yes
Bilge (2022)[36]	Retrospective	Not clear	Turkey	Mean = 46 (11.1)	Screening for those at high risk of breast cancer, staging for newly diagnosed cases, and addressing any abnormalities found in mammography or ultrasound.	1	2	No	338	350 (58)	16.6	81 (47/58)	Distribution and/or IEP**	Histopathology and/or follow-up	Yes
Kwon (2020)[37]	Retrospective	Not clear	Korea	Mean = 52 (30-76)	Not mentioned	1	Not mentioned	Not mentioned	122	126 (100)	79.4	36.5 (46/100)	Distribution and/or IEP**	Histopathology	Not mentioned
Liu (2020)[38]	Not clear	Not clear	China	Unknown for NML	Not mentioned	1	2	Yes	Unknown for NML	50 (32)	64	0 (0/32)	IEP*	Histopathology	Yes
Lv (2022)[39]	Retrospective	Not clear	China	Mean = 51.7	Not mentioned	1	2	Yes	69	94 (27)	28.7	0 (All papillary lesions)	ADC threshold	Histopathology	Not mentioned
Zhao (2020)[40]	Retrospective	Consecutive	China	Mean = 41 (range 21-71)	Not mentioned	2	2	Yes	58	58 (31)	53.4	0 (All IBC)	ADC threshold	Histopathology	Yes
Zhang (2022)[41]	Retrospective	Not clear	China	Unknown for NML detected by MRI	Not mentioned	1	2	No	Unknown for NML detected by MRI	67 (33)	49.2	Unknown for NML detected by MRI	Distribution and/or IEP**	Histopathology	Yes
Li (2023)[42]	Retrospective	Consecutive	China	Malignant = 46.9 (10.4); Benign = 41.4 (10.5)	Not mentioned	1	2	No	343	364 (269)	73.9	12.6 (46/269)	Distribution and/or IEP**	Histopathology	Yes

Yang (2017)[43]	Retrospective	Consecutive	China	Mean = 51.4 (33-79)	1) Insufficient or equivocal mammographic or sonographic findings; 2) Pre-operative staging; 3) Nipple discharge; 4) Palpable breast mass but non-definitive mammography or sonography	1	2	Not mentioned	79	83 (52)	61.9	38.5 (20/52)	Distribution and/or IEP**	Histopathology	Yes
Chen (2021)[44]	Retrospective	Consecutive	Not explicitly mentioned	Mean = 54.1 (27-82)	Screening of high-risk populations, staging, work-up of abnormal imaging on mammography or ultrasound, and treatment-response assessment after neoadjuvant chemotherapy	Not explicitly mentioned	2	Yes	Not mentioned	120 (25)	20.8	84 (21/25)	Distribution and/or IEP**	Histopathology	Yes
Ballesio (2014)[45]	Retrospective	Not clear	Italy	Median = 45 (27-81)	Local staging before surgical treatment, evaluation of the effect of neo-adjuvant chemotherapy, breast already treated for carcinoma, surveillance of high-risk women, patients with nipple discharge, carcinoma of unknown primary syndrome	1	2	No	94	94 (73)	77.7	20.5 (15/73)	Distribution and/or IEP**	Histopathology	Not mentioned

Avendano (2019)[46]	Retrospective	Consecutive	Not explicitly mentioned	Mean = 51.8 (10.8)	Equivocal findings on conventional imaging (BIRADS 0), suspicious lesions or lesions highly suggestive of malignancy on conventional imaging (BIRADS 4 and 5), and pre-operative staging of biopsy-proven breast cancer (BI-RADS 6).	Not explicitly mentioned	2	3	66	66 (39)	59	10.3 (4/39)	ADC threshold	Histopathology	No
Asada (2017)[47]	Retrospective	Not clear	Japan	Not mentioned	Not mentioned	1	2	No	213	213 (178)	83.6	48.9 (87/178)	Distribution and/or IEP**	Histopathology and follow-up	Yes
Bayoumi (2024)[48]	Retrospective	Not clear	Egypt	46.56 ± 10.87	Not mentioned	1	2	Not mentioned	220	220 (166)	75.5	23.5 (39/166)	Distribution and/or IEP** as well as ADC	Histopathology	Not mentioned
Spick (2014)[49]	Retrospective	Consecutive	Austria	Unknown for NML	Following international recommendations, including high-risk screening	1	1	Not mentioned	Unknown for NML	43 (5)	11.6	40 (2/5)	ADC threshold	Histopathology and follow-up	Not mentioned
Tang (2021)[50]	Retrospective	Not clear	China	Benign = 46.88 ± 11.17; Malignant = 54.64 ± 11.12	Not mentioned	1	2	Yes	408	408 (239)	58.6	7.5 (18/239)	ADC threshold	Histopathology	Yes

Clauser (2021)[51]	Retrospective	Not clear	Austria, Germany, the USA, and Italy	Unknown for NML	Not mentioned	5	More than 1	Yes	Unknown for NML	184 (62)	33.7	46.8 (29/62)	ADC threshold	Histopathology	Yes
Jansen (2011)[52]	Retrospective	Consecutive	USA	Unknown for NML	Pre-operative staging of newly diagnosed cancers, postoperative and treatment follow-up, and screening of women at high risk for developing breast cancer	1	1	No	Unknown for NML	261 (212)	81.2	48.6 (103/212)	IEP*	Histopathology	Yes
Li (2023)[53]	Retrospective	Not clear	China	Unknown for NML	Not mentioned	1	2	No	Unknown for NML	45 (27)	60	Unknown for NML	Distribution and/or IEP** as well as ADC	Histopathology	Yes
Cho (2016)[54]	Retrospective	Not clear	Korea	52 (31-75)	Pre-operative staging	1	2	Yes	88	88 (73)	83	95.9 (70/73)	Distribution and/or IEP**	Histopathology	No
Partridge (2010)[55]	Retrospective	Not clear	USA	Unknown for NML	Recently diagnosed breast cancer, high-risk screening, and problem-solving	1	4 fellows	No	Unknown for NML	45 (13)	28.9	Unknown for NML	ADC threshold	Histopathology and follow-up	Yes
Niu (2023)[56]	Retrospective	Consecutive	China	Mean = 49.1 (20-76)	Not mentioned	1	2	No	180	183 (95)	51.9	53.7 (51/95)	Distribution and/or IEP** as well as ADC	Histopathology	Yes
Mohamed	Cross-	Consecutive	Egypt	Mean =	Not mentioned	1	2	No	66	66 (32)	48.5	40.6 (13/32)	Distribution	Histopathology	Not mentioned

(2024)[57]	sectional			41.61 ± 8.7									and/or IEP**		
Ahmadinejad (2024)[58]	Cross-sectional	Not clear	Iran	Mean = 41.7 ± 1.1	Not mentioned	1	2 fellows	No	63	63 (18)	28.6	22.2 (4/18)	Distribution and/or IEP**	Histopathology and follow-up	Yes
Kim (2021)[59]	Retrospective	Consecutive	Korea	55.7 ± 9.59	Pre-operative staging	1	2	Yes	92	93 (55)	59.1	94.5 (52/55)	Distribution and/or IEP**	Histopathology and follow-up	No
Tozaki & Fukuma (2009)[60]	Retrospective	Not clear	Japan	Unknown for NML	Not mentioned	1	1	No	Unknown for NML	44 (28)	63.6	28.6 (8/28)	ADC threshold	Histopathology	Not mentioned
Gity (2014)[61]	Cross-sectional	Consecutive	Iran	Mean = 44.9 ± 8.3 years	Not mentioned	1	1	Yes	188	213 (46)	21.6	Not mentioned	Distribution and/or IEP**	Histopathology and follow-up	Yes
Wilhelm (2012)[62]	Retrospective	Consecutive	USA	Not mentioned	Not mentioned	1	3	Yes	378	578 (141)	24.4	53 (53/141)	Distribution and/or IEP**	Histopathology and follow-up	Not mentioned

MRI, Magnetic resonance imaging; DWI, Diffusion weighted imaging; IEP, Internal enhancement pattern; ADC, Apparent diffusion coefficient; TIC, Time-intensity curve; DCIS, Ductal carcinoma in situ; BI-RADS, Breast Imaging-Reporting and Data System; NME, Non-mass enhancement; US-VAB, Ultrasound-guided vacuum-assisted biopsy; IBC, Invasive breast carcinoma
*IEP: one diagnostic criterion about internal enhancement pattern (clumped enhancement)
**IEP: two diagnostic criteria about internal enhancement pattern (clumped or clustered ring enhancement).

Supplementary Table S4. Diagnostic test parameters of included studies.

Author (Year)	Magnet strength & Manufacturer	Coil types	Enhanced scan sequence & direction	Number of dynamic phases	Slice thickness (mm)	b value (s/mm2)	Maximum lesion diameter	Positioning of the patient
Yabuuchi (2009)[9]	1.5T; Philips	Breast	3D T1-FFE with WATS, coronal	5	1	0, 500, 1000	Malignant lesions = 33.2 (13.9) mm; benign lesions = 26.6 (13.7) mm	Prone
Baltzer (2011)[10]	Not mentioned; Siemens	Not mentioned	2D T1-GE, axial	4	Not mentioned	Not applicable	All lesions = 31.3 (21.4) mm; malignant lesions = 35.2 (23.9) mm; benign lesions = 27.1 (17.7)	Prone
Sakamoto	1.5T; Siemens	Breast	3D FS T1-FLASH, coronal	3	2.5-5	Not applicable	Not mentioned	Prone

(2008)(11)								
Imamura (2010)(12)	1.5T; Philips	Body	3D T1-FFE, coronal	3	2	1000	Not mentioned	Supine
Sotome (2007)(13)	1.5T; GE	Surface	2D FS T1-SPGR, axial	Not mentioned	5	Not applicable	Not mentioned	Prone
Baltzer (2010)(14)	1.5T; Siemens	Phased-array	2D T1- FLASH, axial	7	Not mentioned	Not applicable	The mean diameter of true-positive non-mass lesions was 39.2 ± 29.0 mm	Prone
Goto (2007)(15)	1.5T; Philips	Phased-array	3D FS T1-SPGR, coronal	Not mentioned	3-6	Not applicable	Not mentioned	Prone
Thomassin-Naggara (2011)(16)	1.5T; Philips	Breast	Fs T1-GE, axial	4	1-1.6	Not applicable	Benign = 24.2 +/- 15.3; Malignant = 47.3 +/- 29.1 mm	Prone
Tozaki (2005)(17)	1.5T; Siemens	Breast	3D FS VIBE sequence	3	1.2	Not applicable	Malignant lesions = 41.8 mm (5-110)	Prone
Tokuda (2009)(18)	1.5T; Philips	Surface	3D FS T1-FFE, coronal	4	2/1.5	Not applicable	Not mentioned	Supine
Yuen (2008)(19)	1.5T; Philips	Breast	3D FS T1- TFE, sagittal	3	2	Not applicable	Not mentioned	Prone
Lieberman (2002)(20)	1.5T; GE	Breast	3D FS T1-FSPGR, sagittal	3	2	Not applicable	Not mentioned	Prone
Lieberman (2003)(21)	1.5T; GE	Breast	3D FS T1-FSPGR, sagittal	3	2-3	Not applicable	2 cm (0.7-6.3 cm)	Prone
Di Ninno (2021)(22)	1.5T; GE	Breast	3D FS T1 FSPGR, sagittal	Not mentioned	Not mentioned	Not applicable	Not mentioned	Prone
Moukhtar (2014)(23)	1.5T; GE	Phased-array	3D VIBRANT, axial	Not mentioned	1.2	Not mentioned	Not mentioned	Prone
Liu (2022)(24)	1.5T; Philips	Phased-array	3D T1-FFE, coronal	Not mentioned	2	50 and 1000	Not mentioned	Prone

Aydin (2019)[25]	1.5T; GE	Not mentioned	2D T1 FS GE, axial	Not mentioned	2.8	Not applicable	Median = 23 mm (2-75)	Prone
Zhou (2021)[26]	3T; GE	Breast	3D VIBRANT, not mentioned	Not mentioned	1.2	Not applicable	Malignant = 4.3 (2) cm; Benign = 2.3 (1.9) cm	Not mentioned
Lunkiewicz (2020)[27]	1.5T and 3T; Siemens	Breast	T1 fat-saturated, axial	Not mentioned	1	Not applicable	Not mentioned	Prone
Uematsu & Kasami (2012)[28]	3T; Philips	Breast	3D FS T1 FSPGR, sagittal	6	1	Not applicable	Mean = 35 mm (4-150)	Not mentioned
Chikarmane (2017)[29]	1.5T GE and 3T GE or 3T Siemens	Breast	3D FS T1 FSPGR, sagittal	Not mentioned	Not mentioned	Not applicable	48 mm (20-95) for regional distribution	Not mentioned
Cheng (2013)[30]	1.5T; GE	Phased-array	3D VIBRANT, axial	6	1.2	Not applicable	Unknown for NME	Prone
Marino (2022)[31]	3T; Siemens	Breast	3D FS T1 FSPGR, coronal	Not mentioned	1	Not applicable	Mean = 40 mm (25)	Prone
Kul (2013)[32]	1.5T; Siemens	Breast	3D FS T1 FSPGR, axial	Not mentioned	1	50, 400, and 1000 s/mm2	Unknown for NME	Prone
Yang (2020)[33]	3T; Siemens	Phased-array	FS-CE-T1W-GRE, not mentioned	Not mentioned	1.6	0 and 800	Unknown for NME	Prone
Liu (2023)[34]	1.5T; GE	Surface	3D VIBRANT, not mentioned	Not mentioned	1	0 and 800	Unknown for NME	Not mentioned
Zang (2022)[35]	3T; Siemens	Breast	Not mentioned, axial	5	0.9	0 and 800	Benign = 20.28 (20.64); Malignant = 42 (32.1) mm	Prone
Bilge (2022)[36]	1.5T; GE	Breast	T1-FS, axial	Not mentioned	Not mentioned	Not applicable	19 (5-87) mm	Prone
Kwon (2020)[37]	3T Philips and Siemens	Breast	T1FS, not mentioned	6	Not mentioned	Not applicable	Not mentioned	Prone
Liu (2020)[38]	3T; Siemens	Phased-array	3D FS T1- TFE, sagittal	5	0.9	Not applicable	Not mentioned	Prone
Lv (2022)[39]	3T; Philips	Not mentioned	3D gradient echo FS; not mentioned	4-5	1	0, 800	Not mentioned	Prone

Zhao (2020)(40)	1.5T and 3T; Siemens	Phased-array	3D FS T1 FSPGR, not mentioned	6	Not mentioned	50,400,800	Benign = 60 (14-92); Malignant = 48 (23-105) mm	Prone
Zhang (2022)(41)	3T; GE	Phased-array	3D VIBRANT, axial	9	6	0, 1000	Unknown for NML detected by MRI	Prone
Li (2023)(42)	3T; Siemens	Phased-array	3D TWIST-VIBE	Not mentioned	1.5	0, 50, 1000	Median for invasive = 44.9 (IQR 32-59.7) mm	Prone
Yang (2017)(43)	3T; GE	Phased-array	3D VIBRANT, sagittal	6	1.6	Not applicable	Not mentioned	Prone
Chen (2021)(44)	3T; GE	Breast	3D T1W SPGR; axial	5	Not mentioned	0, 600	For linear NME lesions, the average is 1.8 cm (0.6-7.6 cm)	Prone
Ballesio (2014)(45)	1.5T; Siemens	Breast	3D GRE T1FS; axial	5	1	Not applicable	Not mentioned	Prone
Avendano (2019)(46)	3T; Siemens	Breast	3D T1 VIBES; not mentioned	Not mentioned	Not mentioned	50, 850	Mean = 40 (25) mm	Prone
Asada (2017)(47)	1.5T; Philips	Breast	3D FS T1W FS GRE; axial	4	1.8	Not applicable	Mean = 2.4 cm (0.6–10.2)	Prone
Bayoumi (2024)(48)	1.5T; Not mentioned	Breast	T1W with FS GRE; axial	6	Not mentioned	0, 500 and 1000	Not mentioned	Prone
Spick (2014)(49)	1.5T; Siemens	Breast	2D T1W GE FLASH	7	Not mentioned	50, 400, and 800	Mean = 26.9 ± 15.9 mm (6-67 mm)	Not mentioned
Tang (2021)(50)	1.5T; United Imaging	Breast	3D FS T1W	6	2.4	50 and 800	Not mentioned	Prone
Clauser (2021)(51)	1.5T and 3T; Not mentioned	Breast	Not mentioned	Not mentioned	Not mentioned	Not mentioned	Unknown for NML	Not mentioned
Jansen (2011)(52)	1.5T; GE and Philips (3 machines)	Not mentioned	3D T1W; coronal or axial	3 to 6	2 to 3	Not applicable	Malignant = 36.1 ± 24.8 mm; Benign = 18.9 ± 12.2 mm	Prone
Li (2023)(53)	3T; GE	Breast	T1 FS; not mentioned	8	3	50 and 1000	Unknown for NML	Not mentioned
Cho (2016)(54)	3T; Philips or Siemens	Breast	3D T1W-FS GRE; axial	7	2-3	Not applicable	Malignant = 3.7 ± 1.6; Benign = 3.3 ± 1.5 cm	Prone
Partridge	1.5T; GE	Breast	3D T1WI SPGR with parallel	6	1.6-2.2	0 and 600	Median = 2.5 (0.7-8.3) cm	Not mentioned

(2010)[55]			volume imaging; axial					
Niu (2023)[56]	3T; GE	Breast	T1WI FS; axial	6	1	1000	Mean = 26.1 (5-90) mm	Prone
Mohamed (2024)[57]	1.5T; Siemens	Phased-array	3D FS SPGR T1; axial	8	Not mentioned	50 and 1000	Not mentioned	Prone
Ahmadinejad (2024)[58]	Not mentioned	Not mentioned	T1 FS; axial	Not mentioned	Not mentioned	800	Malignant mean = 48.2 mm, benign = 41.2 mm	Not mentioned
Kim (2021)[59]	3T; GE	Breast	T1WI FS GRE; axial	7	1.6	Not applicable	Benign = 42.8 mm, malignant = 49.9 mm	Prone
Tozaki & Fukuma (2009)[60]	1.5T; Siemens	Breast	3D-FS VIBE; sagittal	4	0.9	500 and 1500	Not available for NME	Not mentioned
Gity (2014)[61]	1.5T; GE	Breast	3D T1W FS GRE; axial	6	4	Not applicable	Not mentioned	Prone
Wilhelm (2012)[62]	1.5T; Siemens	Breast	3D T1W GRE; axial	6	3	Not applicable	Not mentioned	Prone

T, Tesla; 3DFS, 3-dimensional fat saturated; FS, Fat-saturated; T1-FFE, T1-weighted fast field echo; WATS, Water-assisted time series; GE, Gradient echo; SPGR, Spoiled gradient recalled echo; FLASH, Fast low-angle shot; VIBE, Volumetric interpolated breath-hold examination; TFE, Turbo field echo; FSPGR, Fast-spoiled gradient recalled echo; CE-T1W-GRE, Contrast-enhanced T1-weighted gradient echo; GRE, Gradient recalled echo; TWIST, Time-resolved imaging with stochastic trajectories; FS-T1W, Fat-saturated T1-weighted; ADC, Apparent diffusion coefficient; b-value, Diffusion weighting factor.

Supplementary Table S5. Meta-regression analysis of DCE-MRI.

Parameter	Category	Sensitivity	p-value	Specificity	p-value	LRT Chi^2	I^2 (95% confidence interval)	p-value
Number of radiologists	≥2	0.52 [0.33 - 0.70]	0.21	0.72 [0.61 - 0.82]	0.32	2.44	18 (0-100)	0.3
Breast radiologists	Yes	0.56 [0.39 - 0.71]	0.06	0.67 [0.57 - 0.76]	0.03	9.02	78 (52-100)	0.01
Magnet strength	3T	0.80 [0.67 - 0.88]	<0.01	0.68 [0.57 - 0.76]	0.03	40.85	95 (91-99)	<0.01
Coil used	Breast	0.65 [0.50 - 0.78]	<0.01	0.77 [0.69 - 0.84]	0.86	16.73	88 (76-100)	<0.01
Blinding	Yes	0.37 [0.23 - 0.54]	0.88	0.68 [0.57 - 0.77]	0.04	7.24	72 (39-100)	0.03

Country	Asia	0.34 [0.22 - 0.48]	0.54	0.85 [0.78 - 0.89]	0.06	5.66	62 (21-100)	0.06
Direction of scan	Axial	0.55 [0.40 - 0.70]	0.05	0.76 [0.68 - 0.83]	0.68	6.27	68 (29-100)	0.04
Number of dynamic phases	≥6	0.58 [0.43 - 0.72]	0.02	0.83 [0.76 - 0.88]	0.19	17.04	88 (76-100)	<0.01

Supplementary Table S6. Pooled Estimates of Diagnostic Performance of Dynamic Contrast Enhancement of Distribution Pattern.

Parameter	Distribution					
	Segmental	Regional	Linear	Focal	Diffuse	Multiple
No. of studies	29	25	28	24	17	13
No. of lesions	2904	2740	2891	2600	1599	1130
Sensitivity (%)	65 (47-80)	25 (12-43)	28 (13-51)	30 (14-53)	7 (4-12)	10 (4-25)
Specificity (%)	78 (67-86)	82 (76-87)	79 (68-86)	67 (58–75)	94 (87-97)	96 (92-98)
Positive likelihood ratio	2.95 (2.09 – 4.14)	1.3 (0.6 – 3.0)	1.3 (0.7 – 2.3)	0.9 (0.4 – 1.9)	1.2 (0.5-3)	2.5 (0.7-8.6)
Posterior probability (%) assuming a 25% prior probability	50	31	30	23	29	46
Negative likelihood ratio	0.45 (0.29 – 0.69)	0.93 (0.73 – 1.18)	0.92 (0.73 – 1.15)	1.05 (0.76 – 1.43)	0.99 (0.93-1.05)	0.94 (0.84-1.05)
Posterior probability (%) assuming a 25% prior probability	13	24	23	26	25	24
Positive predictive value	0.73 (0.65 – 0.81)	0.57 (0.38 – 0.75)	0.56 (0.36-0.77)	0.48 (0.29 – 0.66)	0.54 (0.33-0.75)	0.7 (0.39-1)
Negative predictive value	0.68 (0.61 -0.74)	0.52 (0.47 – 0.56)	0.52 (0.46-0.58)	0.49 (0.43-0.55)	0.5 (0.47-0.54)	0.52 (0.49-0.54)
The area under the curve	0.79 (0.75 – 0.82)	0.71 (0.67 – 0.75)	0.66 (0.62-0.70)	0.59 (0.54-0.63)	0.32 (0.28-0.36)	0.89 (0.86-0.91)
I^2 (%) and p-value	99 (99-100) and <0.001	97 (96-99) and <0.001	99 (99-100) and <0.001	99 (98-99) and <0.001	96 (92-99) and <0.001	76 (47-100) and 0.008
Publication bias	0.05	0.32	0.03	0.07	0.08	0.64
Likelihood ratio scattergram	RLQ; No exclusion or confirmation	RLQ; No exclusion or confirmation	RLQ; No exclusion or confirmation	RLQ; No exclusion or confirmation	RLQ; No exclusion or confirmation	RLQ; No exclusion or confirmation

RLQ, right lower quadrant.

Supplementary Table S7. Pooled Estimates of Diagnostic Performance of Dynamic Contrast Enhancement of Time-Intensity Curve Pattern.

Parameter	Time Intensity Curve		
	Plateau	Persistent	Washout
No. of studies	20	18	20
No. of lesions	1510	1372	1739

Sensitivity (%)	61 (45-76)	47 (24-72)	59 (31-82)
Specificity (%)	60 (47-71)	50 (37-64)	87 (74-94)
Positive likelihood ratio	1.5 (1 – 2.2)	0.95 (0.6 – 1.6)	4.4 (2.4 – 8.1)
Posterior probability (%) assuming a 25% prior probability	34	24	60
Negative likelihood ratio	0.65 (0.42 – 1.01)	1.04 (0.64 – 1.7)	0.48 (0.26 – 0.88)
Posterior probability (%) assuming a 25% prior probability	18	26	14
Positive predictive value	0.59 (0.51 – 0.68)	0.49 (0.35-0.62)	0.8 (0.7-0.9)
Negative predictive value	0.60 (0.52 -0.67)	0.49 (0.40-0.58)	0.66 (0.60-0.73)
The area under the curve	0.64 (0.59 – 0.68)	0.49 (0.45 – 0.54)	0.84 (0.81-0.87)
I^2 (%) and p-value	98 (96-99) and <0.001	99 (98-99) and <0.001	99 (98-99) and <0.0001
Publication bias	0.54	0.13	0.96
Likelihood ratio scattergram	RLQ; No exclusion or confirmation	RLQ; No exclusion or confirmation	RLQ; No exclusion or confirmation

RLQ, right lower quadrant.

Supplementary Table S8. Pooled Estimates of Diagnostic Performance of Dynamic Contrast Enhancement of Internal Enhancement Pattern.

Parameter	Internal Enhancement Pattern			
	Homogenous	Heterogenous	Clustered Ring	Clumped
No. of studies	27	29	22	29
No. of lesions	3147	3317	1764	3259
Sensitivity (%)	11 (3-30)	55 (41-69)	63 (38-83)	45 (30-61)
Specificity (%)	80 (68-88)	63 (52-74)	84 (66-93)	75 (62–85)
Positive likelihood ratio	0.5 (0.2 – 1.4)	1.5 (1 – 2.2)	3.9 (2.2 – 7)	1.8 (1.1 – 3)
Posterior probability (%) assuming a 25% prior probability	15	33	56	38
Negative likelihood ratio	1.11 (0.98 – 1.27)	0.71 (0.49 – 1.01)	0.44 (0.26 – 0.75)	0.73 (0.55 – 0.98)
Posterior probability (%) assuming a 25% prior probability	27	19	13	20
Positive predictive value	0.36 (0 – 0.75)	0.59 (0.51 – 0.68)	0.78 (0.67-0.89)	0.63 (0.52–0.75)
Negative predictive value	0.48 (0.41 -0.54)	0.58 (0.51 – 0.65)	0.68 (0.60-0.76)	0.57 (0.50-0.65)
The area under the curve	0.57 (0.53 – 0.61)	0.63 (0.59 – 0.67)	0.82 (0.78-0.85)	0.66 (0.61-0.70)
I^2 (%) and p-value	100 (99-100) and <0.001	99 (98-99) and <0.001	99 (99-100) and <0.0001	99 (99-100) and <0.0001
Publication bias	0.41	0.11	0.36	0.07
Likelihood ratio scattergram	RLQ; No exclusion or confirmation	RLQ; No exclusion or confirmation	RLQ; No exclusion or confirmation	RLQ; No exclusion or confirmation

RLQ, right lower quadrant.

Supplementary Table S9. ADC cut-off from each study.

Author (Year)	Apparent diffusion coefficient cut-off (10^{-3} mm^2/s)*
Imamura (2010)[12]	1.1
Liu (2022)[24]	1.3
Kul (2013)[32]	0.9
Yabuuchi (2009)[9]	1.3
Liu (2022)[24]	1.4
Zang (2022)[35]	1.23
Lv (2022)[39]	1.14
Zhao (2020)[40]	0.875
Zhang (2022)[41]	Not mentioned
Li (2023)[42]	0.8295
Avendano (2019)[46]	1.3
Bayoumi (2024)[48]	1.15
Spick (2014)[49]	1.012
Tang (2021)[50]	1.211
Clauser (2021)[51]	1.5
Li (2023)[53]	0.815
Partridge (2010)[55]	1.6
Moukhtar (2014)[23]	1.35
Tozaki & Fukuma (2009)[60]	1.13
Cheng (2013)[30]	1.35
Marino (2022)[31]	1.215

*Values stated indicate that below this threshold, the lesion is more likely malignant.

Supplementary Table S10. Meta-regression analysis of ADC.

Parameter	Category	Sensitivity	p-value	Specificity	p-value	LRT Chi^2	I^2 (95% confidence interval)	p-value
Number of radiologists	≥2	0.71 [0.47 - 0.87]	0.61	0.64 [0.40 - 0.83]	0.81	0.42	0 (0-100)	0.81
Breast radiologists	Yes	0.81 [0.65 - 0.91]	0.50	0.74 [0.56 - 0.86]	0.45	1.6	0 (0-100)	0.45
Magnet strength	3T	0.77 [0.60 - 0.89]	0.87	0.72 [0.54 - 0.85]	0.55	0.57	0 (0-100)	0.75
Coil used	Breast	0.71 [0.52 - 0.85]	0.56	0.66 [0.46 - 0.81]	0.94	0.53	0 (0-100)	0.77
Blinding	Yes	0.87 [0.76 - 0.94]	0.08	0.59 [0.40 - 0.76]	0.45	5.25	62 (14-100)	0.07
Country	Asia	0.79 [0.62 - 0.90]	0.69	0.48 [0.30 - 0.66]	0.05	4.93	59 (9-100)	0.08
Direction of scan	Axial	0.77 [0.57 - 0.89]	0.94	0.82 [0.69 - 0.90]	0.03	5.62	64 (20-100)	0.06
Number of dynamic phases	≥6	0.82 [0.65 - 0.92]	0.48	0.72 [0.53 - 0.86]	0.57	1.26	0 (0-100)	0.53
Cut-off	<1 (10^{-3} mm^2/s)*	0.80 [0.60 - 0.91]	0.70	0.75 [0.56 - 0.88]	0.40	1.23	0 (0-100)	0.54

	<1.5 (10^{-3} mm^2/s)**	0.64 [0.42 - 0.81]	0.19	0.81 [0.66 - 0.91]	0.08	6.28	71 (35-100)	0.03

* Indicated by ADC in Supplementary Figure 1B.**Indicated by ADCX in Supplementary Figure 1B

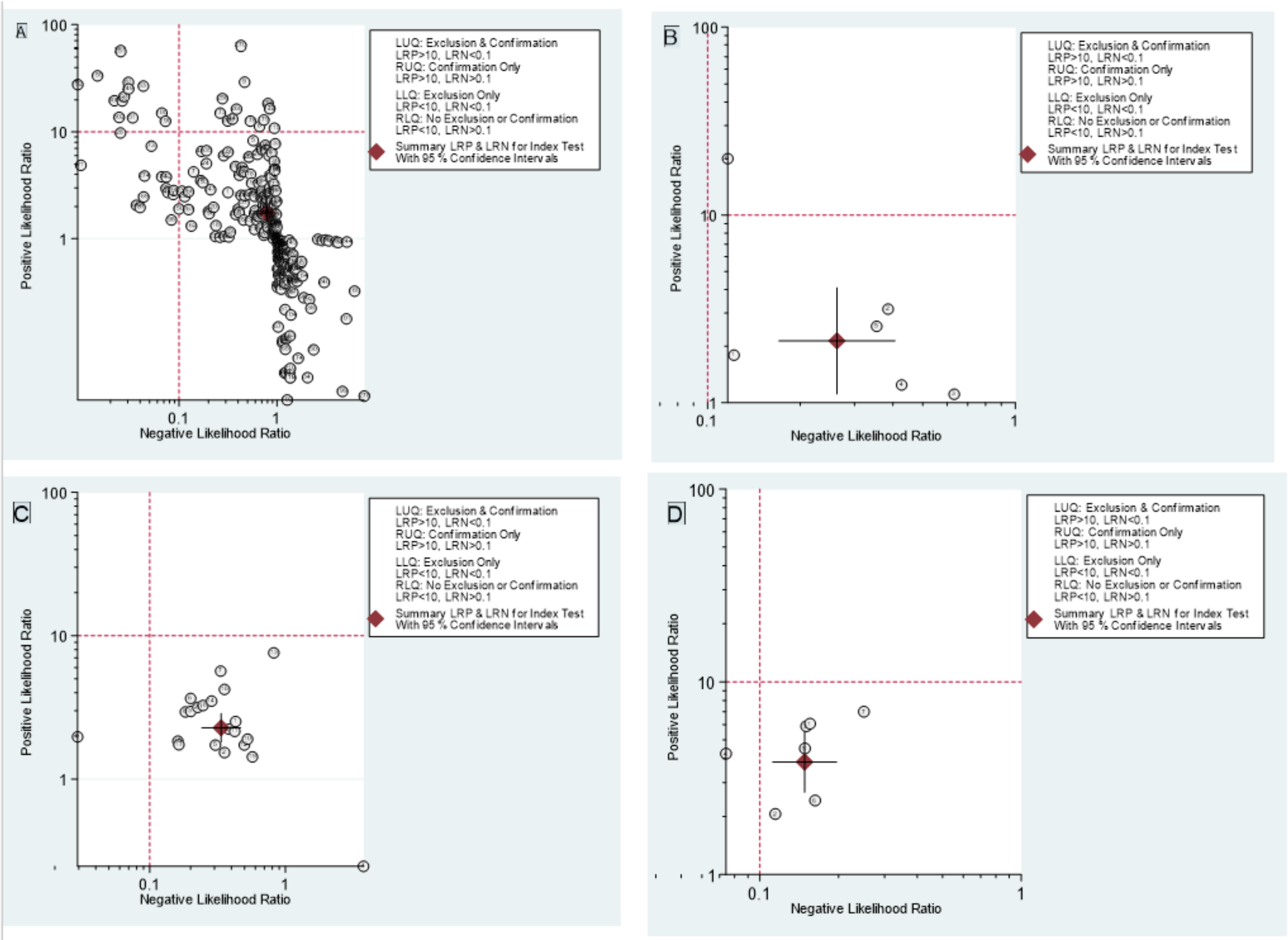

Supplementary Figure S1. Likelihood ratio scattergram of (A) DCE-MRI, (B) DWI, (C) ADC, and (D) DCE+DWI.

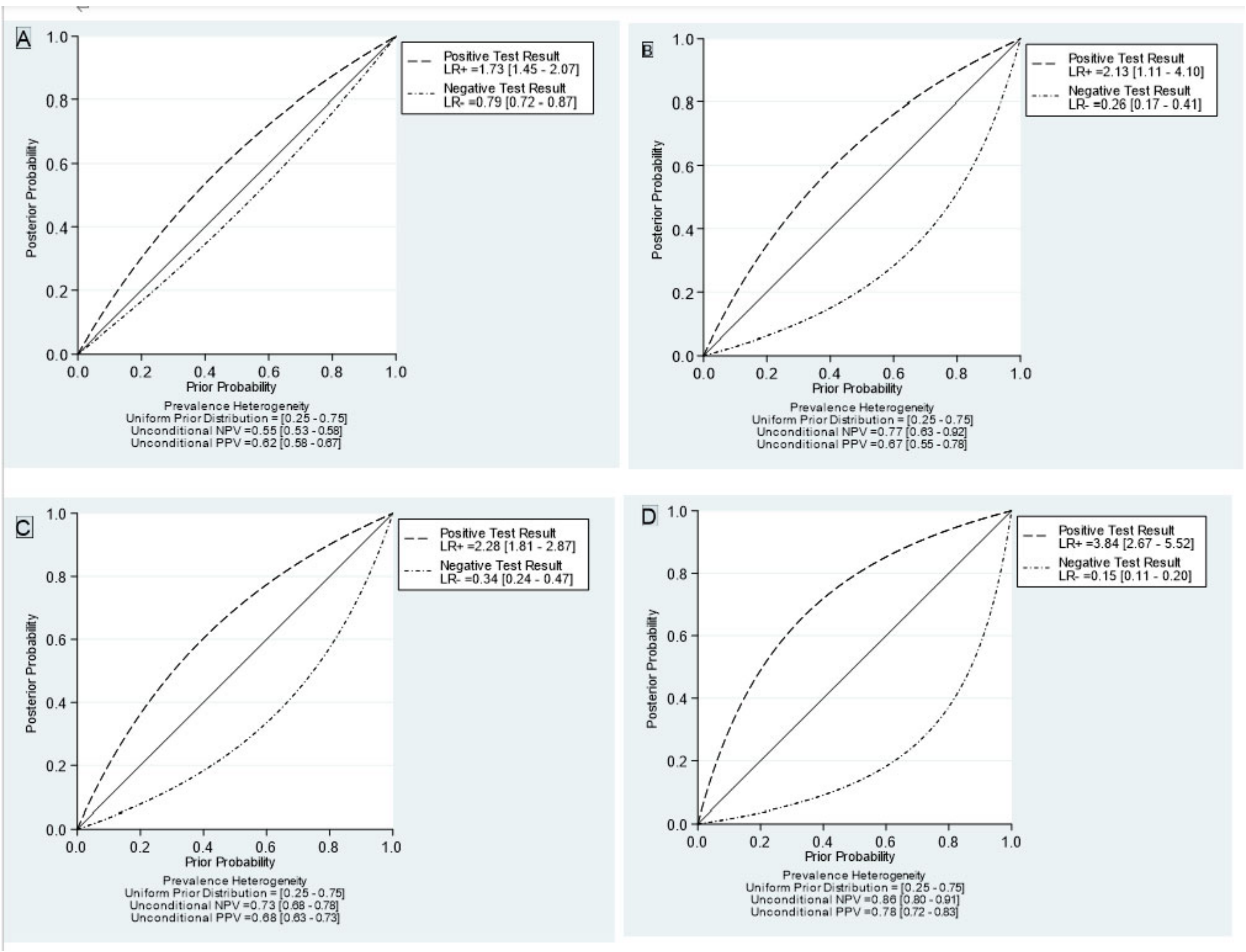

Supplementary Figure S2. Probability Modifying Plot of (A) DCE-MRI, (B) DWI, (C) ADC, and (D) DCE+DWI.

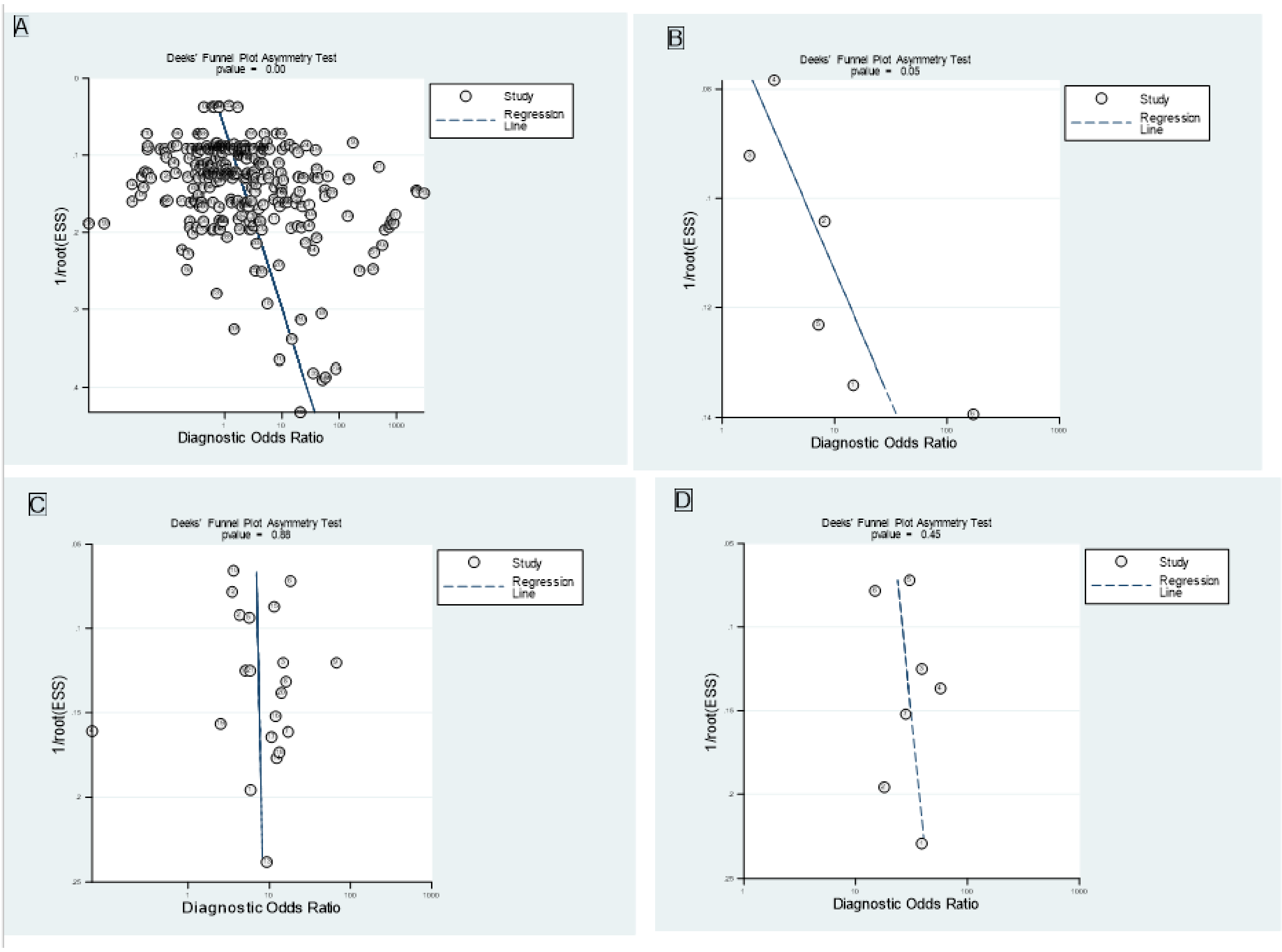

Supplementary Figure S3. Linear regression test of funnel plot asymmetry of (A) DCE-MRI, (B) DWI, (C) ADC, and (D) DCE+DWI.

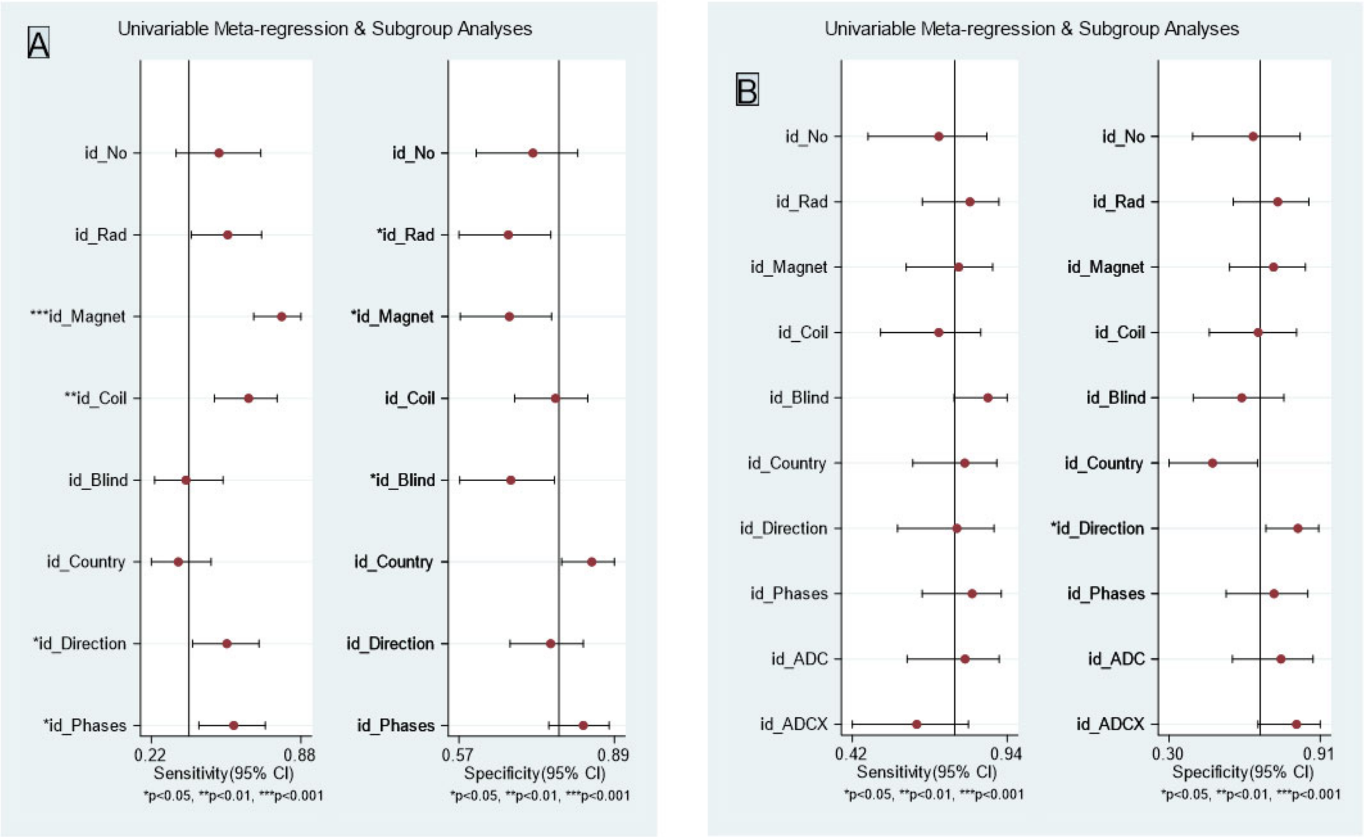

Supplementary Figure S4. Meta-regression of DCE-MRI (A) and ADC (B).

Supplementary References

1. Pinker K, Bickel H, Helbich TH, Gruber S, Dubsky P, Pluschnig U, et al. Combined contrast-enhanced magnetic resonance and diffusion-weighted imaging reading adapted to the "Breast Imaging Reporting and Data System" for multiparametric 3-T imaging of breast lesions. Eur Radiol. 2013;23(7):1791-802.
2. Ei Khouli RH, Jacobs MA, Mezban SD, Huang P, Kamel IR, Macura KJ, et al. Diffusion-weighted imaging improves the diagnostic accuracy of conventional 3.0-T breast MR imaging. Radiology. 2010;256(1):64-73.
3. Shimauchi A, Abe H, Schacht DV, Yulei J, Pineda FD, Jansen SA, et al. Evaluation of Kinetic Entropy of Breast Masses Initially Found on MRI using Whole-lesion Curve Distribution Data: Comparison with the Standard Kinetic Analysis. Eur Radiol. 2015;25(8):2470-8.
4. Santamaría G, Velasco M, Farrús B, Caparrós FX, Fernández PL. Dynamic contrast-enhanced MRI reveals the extent and the microvascular pattern of breast ductal carcinoma in situ. Breast J. 2013;19(4):402-10.
5. Lee SM, Nam KJ, Choo KS, Kim JY, Jeong DW, Kim HY, et al. Patterns of malignant non-mass enhancement on 3-T breast MRI help predict invasiveness: using the BI-RADS lexicon fifth edition. Acta Radiol. 2018;59(11):1292-9.
6. Machida Y, Shimauchi A, Tozaki M, Kuroki Y, Yoshida T, Fukuma E. Descriptors of Malignant Non-mass Enhancement of Breast MRI: Their Correlation to the Presence of Invasion. Acad Radiol. 2016;23(6):687-95.
7. Morakkabati-Spitz N, Leutner C, Schild H, Traeber F, Kuhl C. Diagnostic usefulness of segmental and linear enhancement in dynamic breast MRI. European Radiology. 2005;15(9):2010-7.
8. Parsian S, Rahbar H, Allison KH, Demartini WB, Olson ML, Lehman CD, et al. Nonmalignant breast lesions: ADCs of benign and high-risk subtypes assessed as false-positive at dynamic enhanced MR imaging. Radiology. 2012;265(3):696-706.
9. Yabuuchi H, Matsuo Y, Kamitani T, Setoguchi T, Okafuji T, Soeda H, et al. Non-mass-like enhancement on contrast-enhanced breast MR imaging: lesion characterization using combination of dynamic contrast-enhanced and diffusion-weighted MR images. Eur J Radiol. 2010;75(1):e126-32.
10. Baltzer PA, Dietzel M, Kaiser WA. Nonmass lesions in magnetic resonance imaging of the breast: additional T2-weighted images improve diagnostic accuracy. J Comput Assist Tomogr. 2011;35(3):361-6.
11. Sakamoto N, Tozaki M, Higa K, Tsunoda Y, Ogawa T, Abe S, et al. Categorization of non-mass-like breast lesions detected by MRI. Breast Cancer. 2008;15(3):241-6.

12. Imamura T, Isomoto I, Sueyoshi E, Yano H, Uga T, Abe K, et al. Diagnostic performance of ADC for Non-mass-like breast lesions on MR imaging. Magn Reson Med Sci. 2010;9(4):217-25.
13. Sotome K, Yamamoto Y, Hirano A, Takahara T, Hasegawa S, Nakamaru M, et al. The role of contrast enhanced MRI in the diagnosis of non-mass image-forming lesions on breast ultrasonography. Breast Cancer. 2007;14(4):371-80.
14. Baltzer PA, Benndorf M, Dietzel M, Gajda M, Runnebaum IB, Kaiser WA. False-positive findings at contrast-enhanced breast MRI: a BI-RADS descriptor study. AJR Am J Roentgenol. 2010;194(6):1658-63.
15. Goto M, Ito H, Akazawa K, Kubota T, Kizu O, Yamada K, et al. Diagnosis of breast tumors by contrast-enhanced MR imaging: comparison between the diagnostic performance of dynamic enhancement patterns and morphologic features. J Magn Reson Imaging. 2007;25(1):104-12.
16. Thomassin-Naggara I, Trop I, Chopier J, David J, Lalonde L, Darai E, et al. Nonmasslike enhancement at breast MR imaging: the added value of mammography and US for lesion categorization. Radiology. 2011;261(1):69-79.
17. Tozaki M, Igarashi T, Fukuda K. Breast MRI using the VIBE sequence: clustered ring enhancement in the differential diagnosis of lesions showing non-masslike enhancement. AJR Am J Roentgenol. 2006;187(2):313-21.
18. Tokuda Y, Kuriyama K, Nakamoto A, Choi S, Yutani K, Kunitomi Y, et al. Evaluation of suspicious nipple discharge by magnetic resonance mammography based on breast imaging reporting and data system magnetic resonance imaging descriptors. J Comput Assist Tomogr. 2009;33(1):58-62.
19. Yuen S, Uematsu T, Masako K, Uchida Y, Nishimura T. Segmental enhancement on breast MR images: differential diagnosis and diagnostic strategy. Eur Radiol. 2008;18(10):2067-75.
20. Liberman L, Morris EA, Lee MJ, Kaplan JB, LaTrenta LR, Menell JH, et al. Breast lesions detected on MR imaging: features and positive predictive value. AJR Am J Roentgenol. 2002;179(1):171-8.
21. Liberman L, Morris EA, Dershaw DD, Abramson AF, Tan LK. Ductal enhancement on MR imaging of the breast. AJR Am J Roentgenol. 2003;181(2):519-25.
22. Di Ninno AAM, Mello GGN, Torres US, Shimizu C, Tucunduva TCM, Reis FRS, et al. MRI as a complementary tool for the assessment of suspicious mammographic calcifications: Does it have a role? Clin Imaging. 2021;74:76-83.
23. Moukhtar FZ, Abu El Maati AA. Apparent diffusion coefficient values as an adjunct to dynamic contrast enhanced MRI for discriminating benign and malignant breast lesions presenting as mass and non-mass like enhancement. The Egyptian Journal of Radiology and Nuclear Medicine. 2014;45(2):597-604.
24. Liu G, Li Y, Chen SL, Chen Q. Non-mass enhancement breast lesions: MRI findings and associations with malignancy. Ann Transl Med. 2022;10(6):357.
25. Aydin H. The MRI characteristics of non-mass enhancement lesions of the breast: associations with malignancy. Br J Radiol. 2019;92(1096):20180464.
26. Zhou J, Liu YL, Zhang Y, Chen JH, Combs FJ, Parajuli R, et al. BI-RADS Reading of Non-Mass Lesions on DCE-MRI and Differential Diagnosis Performed by Radiomics and Deep Learning. Front Oncol. 2021;11:728224.
27. Lunkiewicz M, Forte S, Freiwald B, Singer G, Leo C, Kubik-Huch RA. Interobserver variability and likelihood of malignancy for fifth edition BI-RADS MRI descriptors in non-mass breast lesions. Eur Radiol. 2020;30(1):77-86.
28. Uematsu T, Kasami M. High-spatial-resolution 3-T breast MRI of nonmasslike enhancement lesions: an analysis of their features as significant predictors of malignancy. AJR Am J Roentgenol. 2012;198(5):1223-30.
29. Chikarmane SA, Michaels AY, Giess CS. Revisiting Nonmass Enhancement in Breast MRI: Analysis of Outcomes and Follow-Up Using the Updated BI-RADS Atlas. AJR Am J Roentgenol. 2017;209(5):1178-84.
30. Cheng L, Bai Y, Zhang J, Liu M, Li X, Zhang A, et al. Optimization of apparent diffusion coefficient measured by diffusion-weighted MRI for diagnosis of breast lesions presenting as mass and non-mass-like enhancement. Tumour Biol. 2013;34(3):1537-45.
31. Marino MA, Avendano D, Sevilimedu V, Thakur S, Martinez D, Lo Gullo R, et al. Limited value of multiparametric MRI with dynamic contrast-enhanced and diffusion-weighted imaging in non-mass enhancing breast tumors. Eur J Radiol. 2022;156:110523.
32. Kul S, Eyuboglu I, Cansu A, Alhan E. Diagnostic efficacy of the diffusion weighted imaging in the characterization of different types of breast lesions. J Magn Reson Imaging. 2014;40(5):1158-64.
33. Yang X, Dong M, Li S, Chai R, Zhang Z, Li N, et al. Diffusion-weighted imaging or dynamic contrast-enhanced curve: a retrospective analysis of contrast-enhanced magnetic resonance imaging-based differential diagnoses of benign and malignant breast lesions. Eur Radiol. 2020;30(9):4795-805.
34. Liu D, Ba Z, Gao Y, Wang L. Subcategorization of suspicious non-mass-like enhancement lesions(BI-RADS-MRI Category4). BMC Med Imaging. 2023;23(1):182.
35. Zang H, Liu HL, Zhu LY, Wang X, Wei LM, Lou JJ, et al. Diagnostic performance of DCE-MRI, multiparametric MRI and multimodality imaging for discrimination of breast non-mass-like enhancement lesions. Br J Radiol. 2022;95(1136):20220211.
36. Coskun Bilge A, Demir PI, Aydin H, Bostanci IE. Dynamic contrast-enhanced breast magnetic resonance imaging findings that affect the magnetic resonance-directed ultrasound correlation of non-mass enhancement lesions: a single-center retrospective study. Br J Radiol. 2022;95(1132):20210832.
37. Kwon BR, Chang JM, Kim SY, Lee SH, Shin SU, Yi A, et al. Utility and Diagnostic Performance of Automated Breast Ultrasound System in Evaluating Pure Non-Mass Enhancement on Breast Magnetic Resonance Imaging. Korean J Radiol. 2020;21(11):1210-9.

38. Liu W, Zong M, Gong HY, Ling LJ, Ye XH, Wang S, et al. Comparison of Diagnostic Efficacy Between Contrast-Enhanced Ultrasound and DCE-MRI for Mass- and Non-Mass-Like Enhancement Types in Breast Lesions. Cancer Manag Res. 2020;12:13567-78.
39. Lv W, Zheng D, Guan W, Wu P. Contribution of Diffusion-Weighted Imaging and ADC Values to Papillary Breast Lesions. Front Oncol. 2022;12:911790.
40. Zhao Q, Xie T, Fu C, Chen L, Bai Q, Grimm R, et al. Differentiation between idiopathic granulomatous mastitis and invasive breast carcinoma, both presenting with non-mass enhancement without rim-enhanced masses: The value of whole-lesion histogram and texture analysis using apparent diffusion coefficient. Eur J Radiol. 2020;123:108782.
41. Zhang F, Wang J, Jin L, Jia C, Shi Q, Wu R. Comparison of the diagnostic value of contrast-enhanced ultrasound combined with conventional ultrasound versus magnetic resonance imaging in malignant non-mass breast lesions. Br J Radiol. 2023;96(1150):20220880.
42. Li Y, Yang Z, Lv W, Qin Y, Tang C, Yan X, et al. Role of combined clinical-radiomics model based on contrast-enhanced MRI in predicting the malignancy of breast non-mass enhancements without an additional diffusion-weighted imaging sequence. Quant Imaging Med Surg. 2023;13(9):5974-85.
43. Yang QX, Ji X, Feng LL, Zheng L, Zhou XQ, Wu Q, et al. Significant MRI indicators of malignancy for breast non-mass enhancement. J Xray Sci Technol. 2017;25(6):1033-44.
44. Chen ST, Covelli J, Okamoto S, Daniel BL, DeMartini WB, Ikeda DM. Clumped vs non-clumped internal enhancement patterns in linear non-mass enhancement on breast MRI. Br J Radiol. 2021;94(1118):20201166.
45. Ballesio L, Di Pastena F, Gigli S, D'Ambrosio I, Aceti A, Pontico M, et al. Non mass-like enhancement categories detected by breast MRI and histological findings. Eur Rev Med Pharmacol Sci. 2014;18(6):910-7.
46. Avendano D, Marino MA, Leithner D, Thakur S, Bernard-Davila B, Martinez DF, et al. Limited role of DWI with apparent diffusion coefficient mapping in breast lesions presenting as non-mass enhancement on dynamic contrast-enhanced MRI. Breast Cancer Research. 2019;21(1):136.
47. Asada T, Yamada T, Kanemaki Y, Fujiwara K, Okamoto S, Nakajima Y. Grading system to categorize breast MRI using BI-RADS 5th edition: a statistical study of non-mass enhancement descriptors in terms of probability of malignancy. Jpn J Radiol. 2018;36(3):200-8.
48. Bayoumi D, Shokeir FA, Karam R, Elboghdady A. Validity of dynamic contrast-enhanced magnetic resonance imaging of the breast versus diffusion-weighted imaging and magnetic resonance spectroscopy in predicting the malignant nature of non-mass enhancement lesions. Egyptian Journal of Radiology and Nuclear Medicine. 2024;55(1):94.
49. Spick C, Pinker-Domenig K, Rudas M, Helbich TH, Baltzer PA. MRI-only lesions: application of diffusion-weighted imaging obviates unnecessary MR-guided breast biopsies. Eur Radiol. 2014;24(6):1204-10.
50. Tang W, Chen L, Jin Z, Liang Y, Zuo W, Wei X, et al. The diagnostic dilemma with the plateau pattern of the time-intensity curve: can the relative apparent diffusion coefficient (rADC) optimise the ADC parameter for differentiating breast lesions? Clin Radiol. 2021;76(9):688-95.
51. Clauser P, Krug B, Bickel H, Dietzel M, Pinker K, Neuhaus VF, et al. Diffusion-weighted Imaging Allows for Downgrading MR BI-RADS 4 Lesions in Contrast-enhanced MRI of the Breast to Avoid Unnecessary Biopsy. Clin Cancer Res. 2021;27(7):1941-8.
52. Jansen SA, Shimauchi A, Zak L, Fan X, Karczmar GS, Newstead GM. The diverse pathology and kinetics of mass, nonmass, and focus enhancement on MR imaging of the breast. J Magn Reson Imaging. 2011;33(6):1382-9.
53. Li X, Wang H, Gao J, Jiang L, Chen M. Quantitative apparent diffusion coefficient metrics for MRI-only suspicious breast lesions: any added clinical value? Quant Imaging Med Surg. 2023;13(10):7092-104.
54. Cho YH, Cho KR, Park EK, Seo BK, Woo OH, Cho SB, et al. Significance of Additional Non-Mass Enhancement in Patients with Breast Cancer on Preoperative 3T Dynamic Contrast Enhanced MRI of the Breast. Iran J Radiol. 2016;13(1):e30909.
55. Partridge SC, Mullins CD, Kurland BF, Allain MD, DeMartini WB, Eby PR, et al. Apparent diffusion coefficient values for discriminating benign and malignant breast MRI lesions: effects of lesion type and size. AJR Am J Roentgenol. 2010;194(6):1664-73.
56. Niu RL, Li JK, Wang B, Jiang Y, Li SY, Fu NQ, et al. Combination of Breast Ultrasound With Magnetic Resonance Imaging in the Diagnosis of Non-mass-like Breast Lesions Detected on Ultrasound: A New Integrated Strategy to Improve Diagnostic Performance. Ultrasound Med Biol. 2024;50(1):105-11.
57. Mohamed S, Elhamd EA, Attia NM. Non-mass enhancement on breast MRI: Clues to a more confident diagnosis. Egyptian Journal of Radiology and Nuclear Medicine. 2024;55(1):87.
58. Ahmadinejad N, Azizinik F, Khosravi P, Torabi A, Mohajeri A, Arian A. Evaluation of Features in Probably Benign and Malignant Nonmass Enhancement in Breast MRI. Int J Breast Cancer. 2024;2024:6661849.
59. Kim Y, Jung HK, Park AY, Ko KH, Jang H. Diagnostic value of mammography for accompanying non-mass enhancement on preoperative breast MRI. Acta Radiol. 2022;63(8):1032-42.
60. Tozaki M, Fukuma E. 1H MR spectroscopy and diffusion-weighted imaging of the breast: are they useful tools for characterizing breast lesions before biopsy? AJR Am J Roentgenol. 2009;193(3):840-9.
61. Gity M, Ghazi Moghadam K, Jalali AH, Shakiba M. Association of Different MRI BIRADS Descriptors With Malignancy in Non Mass-Like Breast Lesions. Iran Red Crescent Med J. 2014;16(12):e26040.
62. Wilhelm A, McDonough MD, DePeri ER. Malignancy rates of non-masslike enhancement on breast magnetic resonance imaging using American College of Radiology Breast Imaging Reporting and Data System descriptors. Breast J. 2012;18(6):523-6.
